# Estradiol-Induced Modulation of Clindamycin Susceptibility in Mono- and Dual-Species Biofilms of *Lactobacillus gasseri* and *Cutibacterium acnes*: An In Vitro Model Study

**DOI:** 10.3390/microorganisms14061173

**Published:** 2026-05-22

**Authors:** Varvara P. Pavlova, Artem G. Chebotarevskii, Ecaterina V. Diuvenji, Nadezhda A. Loginova, Anna M. Mosolova, Aleksandra S. Novikova, Sergey V. Martyanov, Marina V. Sukhacheva, Alexander I. Netrusov, Andrei V. Gannesen

**Affiliations:** 1 Federal Research Centre “Fundamentals of Biotechnology” of Russian Academy of Sciences, Moscow 117312, Russia; v.pavlova@fbras.ru (V.P.P.); a.chebotarevsky@fbras.ru (A.G.C.); e.duvenzhi@fbras.ru (E.V.D.); n.loginova@fbras.ru (N.A.L.); a.mosolova@fbras.ru (A.M.M.); lady.a.s.novikova@yandex.ru (A.S.N.); s.martyanov@fbras.ru (S.V.M.); sukhacheva@biengi.ac.ru (M.V.S.); 2 Microbiology Department, Lomonosov Moscow State University, Moscow 119234, Russia

**Keywords:** multispecies biofilms, hormones, estradiol, antibiotics, clindamycin, vaginal microbiota, *Lactobacillus gasseri*, *Cutibacterium acnes*

## Abstract

This pilot study investigated the effects of estradiol and clindamycin on mono- and dual-species biofilms of selected reference and clinical isolates of *Lactobacillus gasseri* and *Cutibacterium acnes*, including one vaginal isolate of *C. acnes*. Our findings demonstrate complex, strain-dependent effects of both compounds and their combinations. Estradiol inhibited biofilm formation in *L. gasseri* strains but exhibited divergent impacts on *C. acnes* isolates, stimulating the skin-derived strain while suppressing the vaginal isolate. The observation that pre-adsorbed estradiol tended to enhance its biological activity is consistent with, though does not prove, the hypothesis of a direct hormonal interaction with the bacterial cell envelope. Crucially, estradiol modulated the susceptibility of both species to clindamycin. At the working concentration selected, clindamycin susceptibility varied considerably between strains, with the antibiotic stimulating biofilm growth in skin-derived *C. acnes* HM514 biofilms. In dual-species communities, an apparent inversion of clindamycin activity was observed, suggesting that estradiol may alter antibiotic efficacy in a manner dependent on community composition and strain identity. Furthermore, while transcriptional changes in bacteriocin genes were evident under hormonal and antibiotic pressure, these shifts did not consistently correlate with observed phenotypic antagonistic activity. These results underscore the limitations of traditional mono-species assays and highlight the importance of considering hormonal background, community context, and the substantial phenotypic variability among individual microbial isolates when evaluating antimicrobial interventions.

## 1. Introduction

The human microbiota consists of organ-specific microbial communities that differ markedly in taxonomic composition and ecological structure. Among these, the vaginal microbiota is unique in exhibiting exceptionally low bacterial diversity under healthy conditions, typically being dominated by *Lactobacillus* spp., which may comprise 70–90% of the total microbial community [1]. Minor components of the vaginal microbiota include obligate anaerobes such as *Anaerococcus*, *Corynebacterium*, *Finegoldia*, and *Streptococcus*, as well as opportunistic microorganisms including *Candida* spp., some streptococci, and *Escherichia coli*. The low abundance of these taxa in healthy individuals is largely attributed to the dominance and protective activity of lactobacilli [2,3].

The composition of the vaginal microbiota is dynamic and influenced by multiple host-associated factors, including ethnicity, hormonal status, diet, lifestyle, and genetic background [4,5]. Disruption of this ecological balance leads to dysbiosis and the development of vaginal infections, which are typically associated with increased microbial diversity and an elevation of vaginal pH. Bacterial vaginosis is most commonly linked to the overgrowth of *Gardnerella vaginalis*, *Prevotella bivia*, *Sneathia* spp., and *Atopobium vaginae*, whereas fungal and protozoal vaginoses are associated mostly with *Candida* spp. and *Trichomonas vaginalis*, respectively [6,7].

Based on species composition, the vaginal microbiota has been classified into five community state types (CSTs). Four CSTs are dominated by distinct *Lactobacillus* species, namely *Lactobacillus crispatus* (CST-I), *L. gasseri* (CST-II), *L. iners* (CST-III), and *L. jensenii* (CST-V). In contrast, CST-IV is characterized by reduced lactobacilli and an increased abundance of diverse anaerobes. CST-I and CST-II are generally considered favourable and are associated with optimal vaginal health [8,9]. In particular, CST-II, dominated by *L. gasseri*, is linked to a reduced frequency of colonization by opportunistic and pathogenic microorganisms, reflecting the protective functions of this species, including environmental acidification and production of antimicrobial metabolites [10,11,12].

Advances in next-generation sequencing (NGS) have substantially refined our understanding of the vaginal microbiota, particularly with respect to low-abundance taxa that were previously under-recognized or misclassified using culture-based or targeted molecular methods. One such example is *Cutibacterium acnes*, which for a long time could be incorrectly identified as *Atopobium vaginae*. Re-evaluation using NGS revealed that *C. acnes* is a far more common component of the human microbiota than previously assumed [13]. Although best known as a resident of the skin microbiota, *C. acnes* has also been detected in the oral cavity, gastrointestinal tract, and urogenital tract, indicating a broader ecological distribution [14,15,16]. Recent evidence suggests that *Cutibacterium acnes* may serve as at least a transient component of the vaginal microbiota. For instance, its abundance has been shown to increase in patients following clinical miscarriage [17].

The vaginal microbiota exists predominantly in the form of multispecies biofilms, in which microorganisms adhere to epithelial surfaces or reside within the mucus layer. Biofilm formation facilitates close interactions between microbial cells and the host, contributes to microbiota stability, and plays a central role in protection against invading pathogens [18,19]. At the same time, biofilm organization can increase tolerance to antimicrobial agents and promote the persistence of opportunistic pathogens during treatment.

An additional layer of complexity arises from host-derived hormones, particularly steroid hormones, to which the vaginal microbiota is continuously exposed. Steroid hormones have been shown to modulate bacterial growth, virulence, biofilm formation, and antibiotic susceptibility [20,21,22,23]. For example, estradiol and progesterone stimulate the growth and coaggregation of *Prevotella intermedia* and enhance extracellular polysaccharide matrix production [24]. Similar hormone-dependent effects have been reported for *Staphylococcus aureus*, including alterations in pathogenicity and antibiotic sensitivity [25,26].

In contrast, the effects of steroid hormones on lactobacilli and cutibacteria remain less well characterized. Available evidence suggests that steroid hormones can exert both indirect effects, such as estradiol-induced proliferation of vaginal epithelial cells and increased glycogen availability, and direct effects on bacterial cells, including modulation of adhesion, aggregation, and production of antimicrobial compounds [21,27]. For *C. acnes*, hormone-mediated effects are currently thought to be primarily indirect, for example, through increased lipid availability under androgenic influence [28].

From a pharmacological and clinical perspective, hormone-induced modulation of bacterial physiology is of particular importance because it may alter bacterial susceptibility to antimicrobial therapy. Hormones have been shown to affect the expression of resistance-associated genes, membrane permeability, and metabolic pathways, leading to changes in minimum inhibitory concentrations of antibiotics across multiple bacterial species [29,30,31,32]. However, despite the widespread clinical use of clindamycin and metronidazole for the treatment of vaginal infections, data on the influence of estradiol on the antibiotic susceptibility of vaginal biofilm-forming bacteria remain limited [33].

While *C. acnes* has been detected in the vaginal tract and is generally regarded as an adventitious microorganism at this site, potentially resulting from external introduction or hygiene-related factors, its interaction with *L. gasseri* remains a compelling and under-explored area of research. Furthermore, it is of particular interest to determine whether estradiol exerts a regulatory influence on this microbial association under experimental conditions. Therefore, the aim of this study was to investigate, using an in vitro model, the effect of estradiol on the growth and biofilm formation of *Lactobacillus gasseri* and *Cutibacterium acnes* in mono-species and binary communities, as well as to assess how estradiol modulates their sensitivity to clindamycin.

## 2. Materials and Methods

### 2.1. Strains and Cultivation

Four bacterial strains were used in this study: *Lactobacillus gasseri* ATCC 33323, *L. gasseri* MA4, *Cutibacterium acnes* HM514, and *C. acnes* EAB1.

*L. gasseri* ATCC 33323 (vaginal type strain) was obtained from the Korean Collection of Microorganisms (KCTC, Jeongeup, Republic of Korea). *L. gasseri* MA4 and *C. acnes* EAB1 are vaginal isolates obtained from healthy volunteers and stored in the UNIQEM collection (UQM_41559 and UQM_41544, respectively; UNIQEM, Moscow, Russia). *C. acnes* HM514 is a skin-derived acneic RT5 ribotype strain obtained from the American Type Culture Collection (ATCC, Manassas, VA, USA).

All strains were stored in liquid nitrogen (−196 °C). For experiments, frozen stocks were plated on appropriate agar media to obtain isolated colonies. *L. gasseri* strains were cultured on Mann–Rogosa–Sharpe (MRS) medium, while *C. acnes* strains were cultured on reinforced clostridial medium (RCM) with modification of the media as described previously [26,34]. Solid media cultures were incubated anaerobically at 37 °C for 72 h using sealed GasPak™ (BD, Franklin Lakes, NJ, USA) systems with anaerobic gas-generating sachets Anaerogaz (NIKI-MLT, Saint-Petersburg, Russia). Liquid cultures were grown anaerobically at 37 °C for 72 h in tightly sealed screw-cap centrifuge tubes. In each experiment, cultures were washed twice with sterile PS, cells were resuspended in sterile PS or an appropriate medium, and an appropriate optical density at 540 nm was adjusted.

### 2.2. Active Compounds

Estradiol (Merck, Darmstadt, Germany) was dissolved in 96% ethanol (Donskoy Distillery, Epifan, Russia) and stored at −20 °C in the dark. A physiological concentration of 2.2 × 10^−10^ M was selected based on reported plasma levels in healthy women during the mid-luteal phase. Serial dilutions were prepared to obtain final estradiol concentrations of 2.2 × 10^−10^, 2.2 × 10^−9^, 2.2 × 10^−8^, and 2.2 × 10^−7^ M in the culture medium. The final ethanol concentration in all estradiol-containing samples was 0.06% (*v*/*v*) or approximately 0.01 M (9.4 mM). Additional ethanol-only controls were included in all experiments.

Clindamycin (Hemofarm, Vršac, Serbia) was prepared as a sterile aqueous stock solution (15 µg/mL) by dissolving capsule contents in deionized water, followed by filtration through a 0.22 µm membrane. Aliquots were stored at −20 °C for no longer than 21 days. Final concentrations ranged from 0.001 to 20 µg/mL [35,36].

### 2.3. Biofilms in 96-Well Microtiter Plates

Biofilms were grown in sterile 96-well flat-bottom microtiter plates. The 72 h cultures were harvested by centrifugation, washed twice with sterile physiological saline (PS), and resuspended in fresh MRS or RCM. To achieve stable biofilm growth in microtiter plates, active compounds were administered into the medium, and then an aliquot of prepared cultures was added into the medium to reach the final optical density (OD_540_) = 0.2. Then, 200 µL of the resulting suspension was transferred into each well. Plates were incubated anaerobically at 37 °C for 72 h.

To evaluate the effect of pre-adsorbed estradiol, another independent series of experiments was performed. In microplates, wells were pre-incubated overnight at 37 °C with estradiol solutions in the sterile PS (estradiol pre-adsorbed in the PS, EPAPS), 96% ethanol (estradiol pre-adsorbed in ethanol, EPAE) or sterile MRS (estradiol pre-adsorbed in the MRS, EPAMRS). After pre-adsorption, liquids were removed; wells were washed once with PS and subsequently inoculated with media containing appropriate bacterial suspensions and active compounds. Biofilms formed under standard estradiol administration served as controls.

After incubation, wells were washed with PS to remove planktonic cells. Biofilms were fixed with 96% ethanol for 20 min and stained with 0.1% crystal violet following a standard protocol [37].

### 2.4. Static Growth Kinetics Study

Experiments were conducted in 96-well plates (Wuxi NEST Biotechnology, Wuxi, China), cultures and active compounds were prepared and mixed as standard. After plating, 100 µL of sterile vaseline oil (Tula Pharmaceutical Factory, Tula, Russia). was added to each well to limit gas exchange. Edges of the plate were sealed with plasticine and coated with vacuum grease. The plate was pre-incubated in a sealed GasPak vacuum bag for 30 min in the presence of an Anaerogaz anaerobic gassing bag to remove oxygen. After this pre-conditioning step, the bags were hermetically sealed. Cultivation was carried out in a plate spectrophotometer-incubator (XMark Biorad, Hercules, CA, USA; or Allsheng Feyond A300, Hangzhou Allsheng Instruments Co., Hangzhou, China) at 37 °C for 72 h with no shaking. Automatic optical density measurements at 540 nm were taken every 20 min to plot kinetic growth curves.

The resulting kinetic growth curves were analyzed in Microsoft Excel (Microsoft Corporation, Redmond, WA, USA) as described previously [38]. Averaged curves across all replicates were plotted for each experimental group. The specific growth rate (μ, h^−1^) and generation time (g, h) were calculated from semilogarithmic plots of ln(OD) versus time. The exponential growth period was identified by the region with the maximum slope of the linear equation, calculated using the “Slope” function. Kinetic parameters were determined using standard formulas based on the obtained coefficients.

### 2.5. Biofilms on Glass Fiber Filters

Colony-forming unit (CFU) enumeration was performed in parallel with MTT staining of biofilms with minor modifications to a previously described protocol [39]. To reduce variability in inoculation, glass fiber filters were blotted six times with standardized bacterial suspensions in sterile PS (OD_540_ = 1). Biofilms were grown for 72 h on glass fiber filters (Whatman GF/F, 21 mm in diameter, Cytiva, Marlborough, MA, USA) placed on MRS agar supplemented with test compounds and their mixtures.

For CFU determination, filters were transferred to tubes containing 10 mL of PS, mechanically disrupted by trituration and vortexing for 60 s, serially diluted (10^3^–10^5^), and plated (10 µL) onto appropriate media (MRS for *L. gasseri*; RCM for *C. acnes*). In binary biofilms, suspensions were plated on both media to enumerate each species separately. Plates were incubated anaerobically at 37 °C.

Biofilm metabolic activity was assessed using the MTT assay. Filters were incubated with 0.1% MTT (Merck, Darmstadt, Germany) in LB medium (Diam, Moscow, Russia) for 30 min at room temperature, washed, and formazan was extracted with dimethyl sulfoxide (JSC EKOS-1, Moscow, Russia). Absorbance was measured at 540 nm [40,41].

### 2.6. Analysis of Antibacterial Activity

To evaluate the potential antagonistic effects (antibacterial activity) of the studied microorganisms against each other, the agar block diffusion method was employed [42].

To create standard mono-species lawns, standardized suspensions of 72 h cultures of *C. acnes* and *L. gasseri* were used (OD_540_ = 4). A 0.5 mL aliquot of the suspension was applied to the surface of MRS agar (for lactobacilli) or RCM agar (for cutibacteria) with active compounds and spread using a sterile spatula. The plates were then incubated under anaerobic conditions at 37 °C for 72 h.

Aerobic test bacteria *Staphylococcus epidermidis* ATCC 14990, *Micrococcus luteus* C01, *Pseudomonas aeruginosa* PAO1, *Candida albicans* ATCC 10231, and *Staphylococcus aureus* 209P were cultivated for 24 h at 33 °C aerobically in the LB medium. For the pour plate method, 100 µL of a standardized test bacterium suspension (OD_540_ = 0.1) was added to 20 mL of molten and chilled LB-agar (45–50 °C). After thorough mixing, the medium was poured into Petri dishes to ensure a uniform distribution of microorganisms throughout the agar depth. To assess the antagonism between strains of *L. gasseri* and *C. acnes*, 100 µL of an OD_540_ = 2 culture was administered into molten RCM (*C. acnes*) or MRS (*L. gasseri*).

Agar blocks were cut as described previously [42]. Briefly, 4 mm blocks were cut from the lawns using a sterile syringe. These blocks were placed onto the surface of the agar containing the pour-plated cultures. The plates were then incubated under anaerobic conditions at 37 °C for 72 h for *C. acnes* or *L. gasseri* pour plates, or at 33 °C for 24 h for aerobic microorganisms, to assess antimicrobial activity.

### 2.7. In Situ Detection of C. acnes and L. gasseri Bacteriocin Genes and Primer Selection

Bacteriocin genes (cutimycin and acnecins) were identified based on published genome annotations. Primers were designed using Primer-BLAST (NCBI, Bethesda, MD, USA, Primer3 2.5.0) [43] and evaluated for hairpin formation and self-dimerization using OligoAnalyzer™ (IDT) [44]. Primer sequences are provided in Supplementary Materials Table S1. The 16S rRNA gene was used as a reference gene. Corresponding 16S rRNA gene sequences were retrieved from the NCBI database.

Primer specificity for *C. acnes* and *L. gasseri* target sequences was verified by conventional PCR. Reactions were prepared using the M-435 kit (Synthol, Moscow, Russia) in a final volume of 25 µL containing 2.5 µL dNTPs (2.5 mM), 2.5 µL 10 × PCR buffer, 2.5 µL MgCl_2_ (25 mM), 0.2 µL SynTaq DNA polymerase, 0.1 µL TaqMan probe, 1 µL DNA template, and 0.5 µL each of forward and reverse primers (10 pmol/µL), with nuclease-free water added to volume.

PCR amplification was performed with an initial denaturation at 95 °C for 5 min, followed by 25 cycles of 95 °C for 30 s and 60–62 °C for 40–50 s, and a final hold at 4 °C. Amplification products were resolved by electrophoresis in 1% agarose (Diam, Moscow, Russia) gels prepared in 1× TAE buffer (160 V, 20 min), stained with ethidium bromide, and visualized under UV illumination using Gel Doc XR instrument in Quantity One software (version 4.6.5, build 094, Bio-Rad Laboratories, Inc., Hercules, CA, USA).

### 2.8. Differential Expression Analysis of Bacteriocin Genes in L. gasseri and C. acnes via Reverse Transcription PCR (RT-PCR)

For gene expression analysis, biofilms were cultured on the surface of cellulose filters as described previously [45]. Due to the specificities of the EAB1 strain, it was cultivated in liquid medium using glass fiber filters (6 mm diameter) as carriers placed in 96-well plates. Liquid MRS medium supplemented with the test compounds was inoculated with bacterial suspensions to a final OD_540_ of 0.2 and incubated anaerobically at 37 °C for the time required for biofilm formation.

Total RNA was isolated using a phenol–chloroform extraction method combined with mechanical disruption. Biofilm biomass on filters was lysed in the presence of quartz sand by adding 500 µL phenol and 500 µL SNE lysis buffer, followed by homogenization in a FastPrep (MP Biomedicals, Irvine, CA, USA) instrument (six cycles of 20 s with cooling on ice). Samples were vortexed for 15 s, incubated at room temperature for 3 min, and centrifuged at 12,000× *g* for 15 min at 4 °C. The aqueous phase was subjected to two rounds of chloroform extraction, and RNA was precipitated with isopropanol, washed twice with 75% ethanol, air-dried, and resuspended in nuclease-free water. RNA concentration and purity were assessed spectrophotometrically, and integrity was verified by denaturing agarose gel electrophoresis.

cDNA synthesis was performed using MMLV reverse transcriptase (Evrogen, Moscow, Russia). Quantitative RT-PCR was carried out using the M-435 master mix (Synthol, Moscow, Russia) with SYBR Green I and ROX passive reference on a CFX96 Touch real-time PCR system (Bio-Rad, Hercules, CA, USA). Reactions were run in duplicate under the following conditions: initial denaturation at 95 °C for 5 min, followed by 40 cycles of 95 °C for 15 s, 55 °C for 20 s, and 62 °C for 40 s. Negative controls without template (ddH_2_O) were included in each run.

### 2.9. Experimental Design and Data Processing

Data presentation varied depending on the assay type. For biofilm quantification (CV and MTT assays), the results were expressed as relative values normalized to specific reference groups. Specifically, clindamycin-treated groups were compared to untreated controls. Samples containing estradiol or an ethanol-clindamycin combination were normalized to ethanol-treated controls. The estradiol-clindamycin combination group was compared to the estradiol-only group.

For CFU counts, antibacterial activity, kinetics, and gene expression analyses, absolute values were used, with untreated samples serving as the baseline control. In experiments involving pre-adsorbed estradiol, ESA samples served as controls for EPAPS, EPAE, and EPAMRS, while ESA-clindamycin samples were used as references for their respective clindamycin-treated counterparts.

### 2.10. Statistics

All experiments were performed in at least three independent biological replicates. The experiments in 96-well plates were performed four times. The biofilm experiments on filters were performed six times. RNA isolation and differential gene expression experiments, as well as pre-adsorption of estradiol and antibacterial activity tests, were conducted in three independent replicates. Growth kinetics experiments were performed four times. Statistical analyses were performed using GraphPad Prism v. 8.3.1 (GraphPad Software, Boston, MA, USA). Differences between groups were evaluated using the Mann–Whitney U test. Data were normalized to appropriate control groups depending on treatment. The histograms represent individual data points, medians, and the full range from maximum to minimum values, with the exception of MTT assay plots, where individual points were omitted. For gene expression and antibacterial activity data, the results are presented as mean values. In these experiments, the significance of differences was determined using multiple Mann–Whitney U tests. The results were considered statistically significant at *p* < 0.05.

The lack of multiplicity adjustment, such as the Benjamini–Hochberg or Bonferroni correction, may be viewed as a weakness of the statistical approach. However, the number of independent experiments was relatively small, and each experiment included a limited number of comparison groups. Specifically, the ethanol-treated samples and the antibiotic-treated samples were compared against the untreated control. The estradiol-treated samples and the samples treated with the combination of ethanol and clindamycin were compared against the ethanol-treated samples. The samples treated with the combination of estradiol and clindamycin were compared against the samples treated with clindamycin and ethanol. Furthermore, we aimed to avoid type II errors.

## 3. Results

### 3.1. Biofilms in Microtiter Plates

#### 3.1.1. Dose-Dependent Effects of Estradiol on Mono-Species Biofilms

We first tested a wide range of estradiol concentrations to determine the hormone level that significantly affected biofilm formation in *L. gasseri* and *C. acnes* strains.

**Figure 1:**
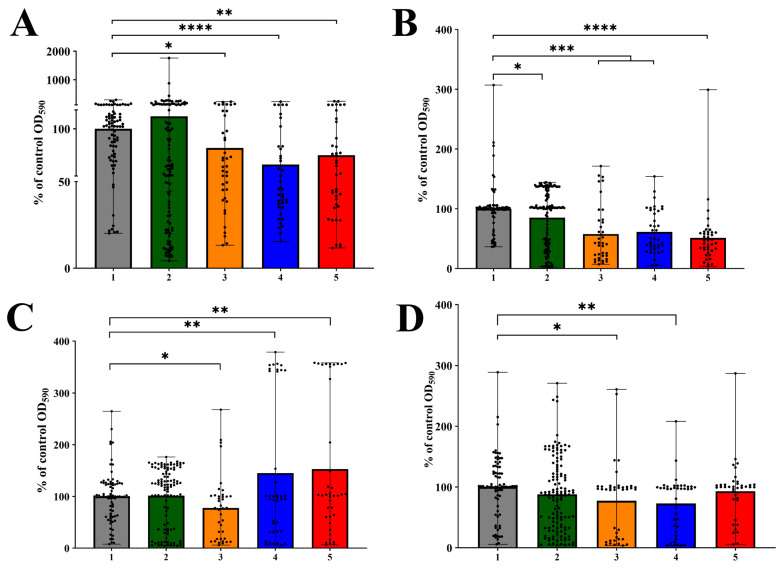
Effect of estradiol in different concentrations on the growth of mono-species biofilms stained with the CV: (**A**)—*L. gasseri* ATCC 33323; (**B**)—*L. gasseri* MA4; (**C**)—*C. acnes* HM514; (**D**)—*C. acnes* EAB1. 1—ethanol 0.01 M; 2—estradiol 2.2 × 10^−10^ M; 3—estradiol 2.2 × 10^−9^ M; 4—estradiol 2.2 × 10^−8^ M; 5—estradiol 2.2 × 10^−7^ M. * means *p* < 0.05, ** means *p* < 0.01; *** means *p* < 0.005; **** means *p* < 0.001. Statistical analysis was performed using the Mann–Whitney U test.

Crystal violet staining revealed a clear correlation between biofilm growth rate and hormone concentration in the culture medium. After 72 h of incubation, estradiol significantly inhibited biofilm formation in both *L. gasseri* strains in a dose-dependent manner (Figure 1A,B). For the *L. gasseri* ATCC 33323 strain, inhibition was already observed at the 10 × physiological concentration (2.2 × 10^−9^ M, Figure 1A). The inhibition effect grew with the hormone concentration, reaching a maximum (62.17% of the control value, *p* < 0.001) at a concentration of 2.2 × 10^−8^ M. At the physiological concentration, there was no pronounced effect. A similar dose-dependent effect was shown for the *L. gasseri* MA4 strain, where the maximum suppression of up to 51.22% of the control was observed at a concentration of 2.2 × 10^−7^ M, whereas at the physiological concentration (2.2 × 10^−10^ M) the inhibition was 19.77% (*p* < 0.05).

In *C. acnes* (Figure 1C,D)*,* different and controversial dose-dependent effects of estradiol were revealed. In the skin strain *C. acnes* HM514, estradiol stimulated biofilm growth. At the highest tested concentration, the hormone increased growth to 152.7% (*p* < 0.05) of the control, while at 2.2 × 10^−9^ M, there was 23% inhibition (*p* < 0.05). In contrast, vaginal strain EAB1 was mostly inhibited in the presence of the hormone. The most significant inhibition of *C. acnes* EAB1 biofilms (25.07%) was observed at the lowest estradiol concentration (2.2 × 10^−10^ M, *p* < 0.05), while at the highest concentration, as well as at the physiological concentration, there was no inhibition.

Based on these data, a physiological hormone concentration (2.2 × 10^−10^ M) was selected for subsequent experiments. The selection criteria were: the presence of a significant suppressive effect or a slight tendency to inhibit biofilm growth in the majority of the tested strains at a given concentration and its correspondence to the level recorded in human blood plasma. Since direct measurement of tissue concentration of the hormone is associated with methodological difficulties, its concentration in blood plasma was chosen as the working baseline.

#### 3.1.2. Dose-Dependent Effects of Clindamycin on Mono-Species Biofilms

All strains used in this study can be considered clinically relevant. Specifically, the minimum inhibitory concentration (MIC) of clindamycin is 10 µg/mL for *L. gasseri* ATCC 33323 and 20 µg/mL for *L. gasseri* MA4. Both cutibacterial strains exhibit MIC values exceeding 20 µg/mL.

**Figure 2:**
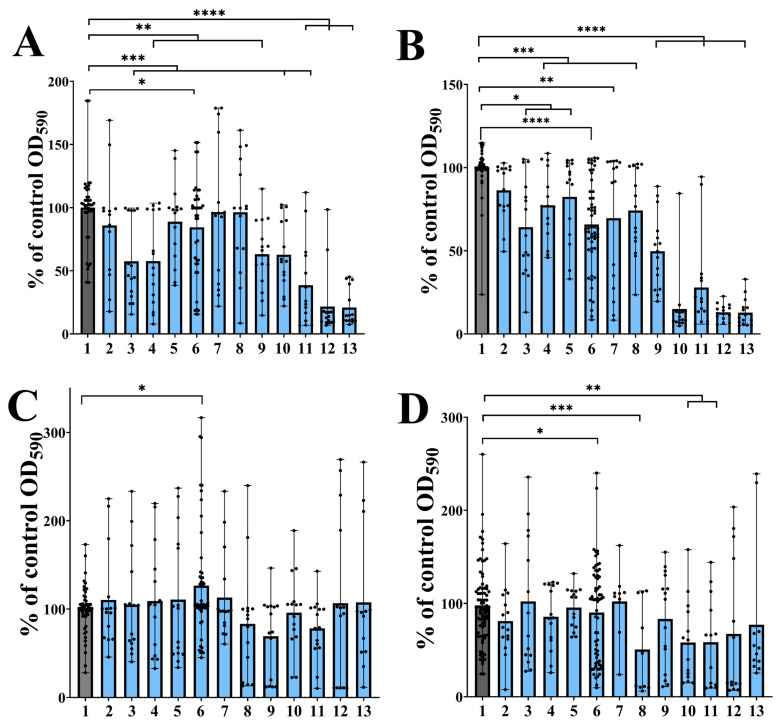
Effect of clindamycin in different concentrations on the growth of mono-species biofilms stained with the CV: (**A**)—*L. gasseri* ATCC 33323; (**B**)—*L. gasseri* MA4; (**C**)—*C. acnes* HM514; (**D**)—*C. acnes* EAB1. Clindamycin concentrations: 1—control; 2—0.001 µg/mL; 3—0.01 µg/mL; 4—0.05 µg/mL; 5—0.1 µg/mL; 6—0.5 µg/mL; 7—1 µg/mL; 8—2 µg/mL; 9—4 µg/mL; 10—6 µg/mL; 11—8 µg/mL; 12—10 µg/mL; 13—20 µg/mL. * means *p* < 0.05, ** means *p* < 0.01; *** means *p* < 0.005; **** means *p* < 0.001. Statistical analysis was performed using the Mann–Whitney U test.

Clindamycin (0.001–20 µg/mL) showed pronounced, dose-dependent inhibition of *L. gasseri* biofilms (Figure 2). In *L. gasseri* ATCC 33323, significant inhibition began at 4 µg/mL (33.86% inhibition, *p* < 0.005) and increased with concentration, reaching 85.55% inhibition at 20 µg/mL (*p* < 0.001; Figure 2A). It is interesting that the inhibition was also at 0.5 µg/mL (14%, *p* < 0.05). *L. gasseri* MA4 was more susceptible: inhibition was significant already at 0.01 µg/mL (59.22%, *p* < 0.05), and biofilm formation was nearly abolished at 20 µg/mL (Figure 2B).

*C. acnes* strains differed in susceptibility. HM514 showed no inhibition across the tested range (Figure 2C), consistent with the presence of clindamycin-resistant *C. acnes* lineages [46]. At 0.5 µg/mL, there was even a stimulation (126%, *p* < 0.05, Figure 2C). EAB1 showed moderate sensitivity, with significant inhibition beginning at 2 µg/mL (29.17%, *p* < 0.005), without a clear linear dose–response thereafter (Figure 2D). Overall, the vaginal isolates (*L. gasseri* MA4 and *C. acnes* EAB1) tended to be more susceptible than the collection strains.

### 3.2. Static Growth Kinetic Study of L. gasseri and C. acnes

To evaluate effects on growth dynamics, OD-based kinetic curves were generated and exponential-phase parameters (*μ* and generation time, *g*) were calculated. Kinetic analysis revealed that estradiol and clindamycin modulate microbial proliferation in a highly strain-specific and dose-dependent manner. While *L. gasseri* strains showed varied sensitivity to physiological estradiol, ranging from mild inhibition (ATCC 33323) to biphasic responses (MA4), *C. acnes* isolates exhibited divergent trends, with estradiol partially antagonizing ethanol-induced suppression in the skin-derived HM514 but exacerbating inhibition in the vaginal isolate EAB1. Both *L. gasseri* strains were highly susceptible to clindamycin, whereas *C. acnes* demonstrated significantly higher tolerance, particularly the HM514 strain. Detailed kinetic parameters, including specific growth rates (*μ*), generation times, and corresponding growth curves (are provided in Supplementary Materials Section S2 and Figures S1 and S2.

### 3.3. Biofilm Growth on Estradiol-Modified Polystyrene Surfaces

**Figure 5:**
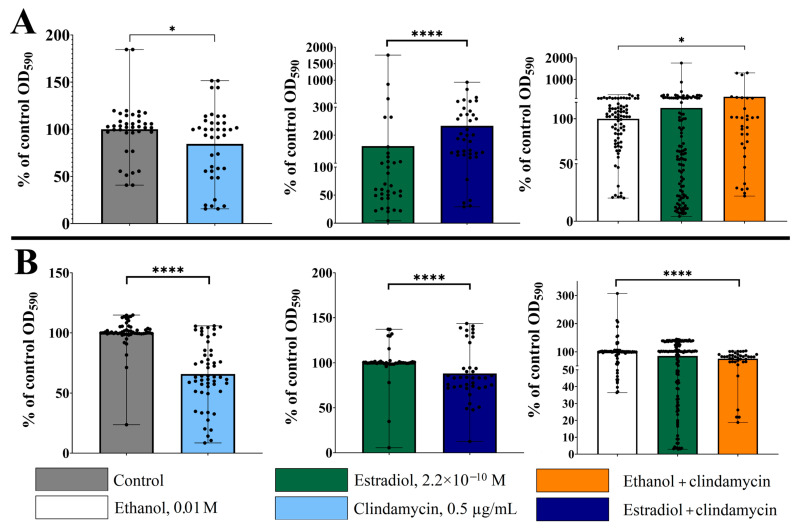
Effects of estradiol (2.2 × 10^−10^ M), clindamycin (0.5 μg/mL), and their combination on the growth of mono-species biofilms of lactobacilli. (**A**)—*L. gasseri* ATCC 33323; (**B**)—*L. gasseri* MA4. * denotes *p* < 0.05; **** denotes *p* < 0.001. Statistical analysis was performed using the Mann–Whitney U test.

**Figure 6:**
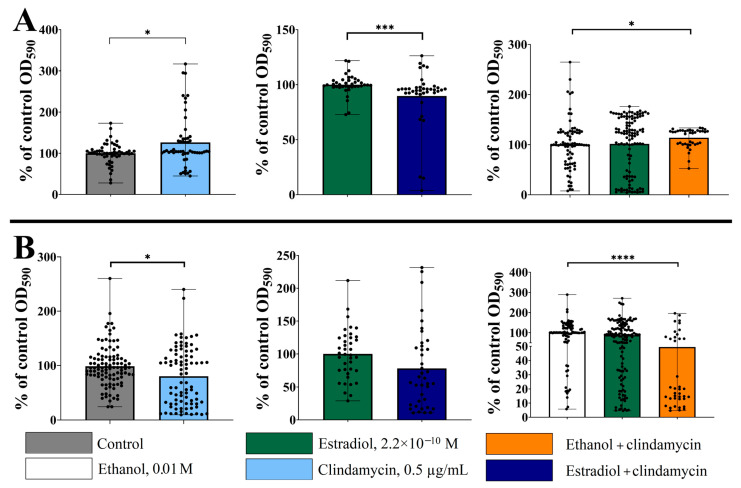
Effects of estradiol (2.2 × 10^−10^ M), clindamycin (0.5 μg/mL), and their combination on the growth of *C. acnes* biofilms. (**A**)—*C. acnes* HM514; (**B**)—*C. acnes* EAB1. * denotes *p* < 0.05; *** denotes *p* < 0.005; **** denotes *p* < 0.001. Statistical analysis was performed using the Mann–Whitney U test.

To assess the contribution of estradiol adsorption to hydrophobic surfaces, biofilm formation was compared between estradiol standard administration (ESA) and estradiol pre-adsorbed from physiological saline, ethanol, or MRS medium (EPAPS, EPAE, EPAMRS). Overall, the administration mode did not produce statistically significant differences in most systems; however, pre-adsorbed estradiol consistently showed an insignificant tendency toward stronger biofilm stimulation (Figures 5 and 6), independent of the solvent used for adsorption.

**Figure 3:**
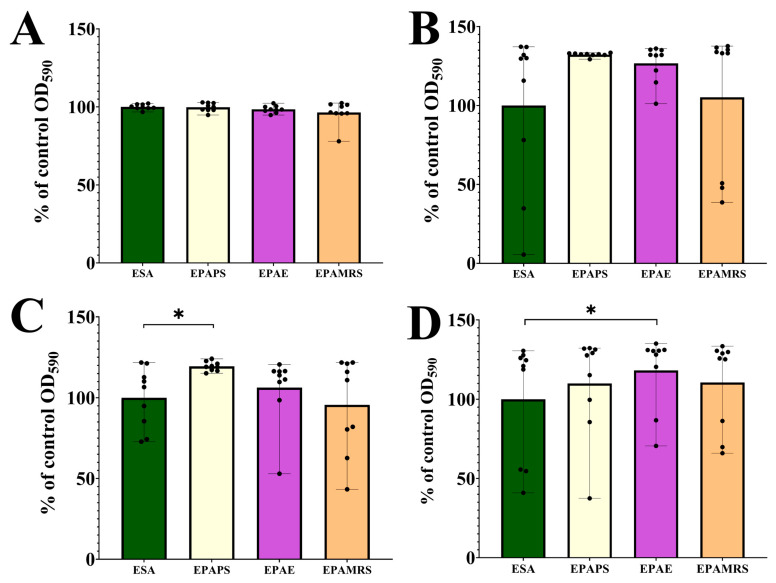
Effects of estradiol, administered via standard method or pre-adsorbed onto the well surface, on mono-species biofilms. (**A**)—*L. gasseri* ATCC 33323; (**B**)—*L. gasseri* MA4; (**C**)—*C. acnes* HM514; (**D**)—*C. acnes* EAB1. ESA: Estradiol Standard Administration; EPAPS: Estradiol Pre-Adsorption from Physiological Saline; EPAE: Estradiol Pre-Adsorption from Ethanol; EPAMRS: Estradiol Pre-Adsorption from MRS medium. * denotes *p* < 0.05. Statistical analysis was performed using the Mann–Whitney U test.

In mono-species biofilms, EPAPS increased *C. acnes* HM514 biofilm formation by 19.4% compared to the non-adsorbed control (*p* < 0.05; Figure 3C). A comparable stimulatory effect of 18.1% was observed for *C. acnes* EAB1 EPAE samples (*p* < 0.05; Figure 3D). *L. gasseri* MA4 behaved similarly to C. acnes HM514; however, its reaction to estradiol was less stable and did not have statistical significance (Figure 3B). In contrast, *L. gasseri* ATCC 33323 biofilms responded similarly to estradiol regardless of the administration method.

**Figure 4:**
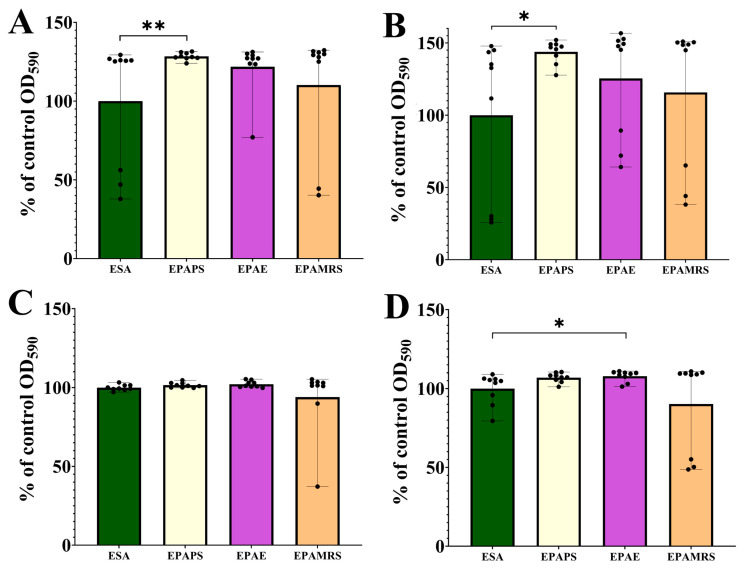
Effects of estradiol, administered via standard method or pre-adsorbed onto the well surface, on dual-species biofilms. (**A**)—*L. gasseri* ATCC 33323 and *C. acnes* HM514; (**B**)—*L. gasseri* ATCC 33323 and *C. acnes* EAB1; (**C**)—*L. gasseri* MA4 and *C. acnes* HM514; (**D**)—*L. gasseri* MA4 and *C. acnes* EAB1. ESA: Estradiol Standard Administration; EPAPS: Estradiol Pre-Adsorption from Physiological Saline; EPAE: Estradiol Pre-Adsorption from Ethanol; EPAMRS: Estradiol Pre-Adsorption from MRS medium. * denotes *p* < 0.05; ** denotes *p* < 0.01. Statistical analysis was performed using the Mann–Whitney U test.

Dual-species biofilms largely mirrored mono-species trends. A significant increase in biofilm biomass was observed for both *L. gasseri* ATCC 33323—*C. acnes* communities (28%, *p* < 0.01 with HM514 strain and 43%, *p* < 0.05 with EAB1 strain, Figure 4A,B respectively), following estradiol pre-adsorption from physiological saline (Figure 4B). The EPAE samples also demonstrated stimulatory trends of lower significance. However, in *L. gasseri* MA4—*C. acnes* EAB1 community statistically significant difference in small magnitude (7%) was detected in the EPAE system, while EPAPS samples were out of statistical significance (Figure 4D), likely reflecting high data consistency rather than biologically meaningful effects. In the MA4—HM514 community, and in each EPAMRS, no significant timulatory trends were observed.

Taken together, estradiol pre-adsorption modestly stimulated both mono-species and dual-species biofilms, with physiological saline providing the most reproducible adsorption conditions, consistent with previous observations in mixed-species biofilms of *S. aureus* and *L. paracasei* [26].

### 3.4. The Effect of Estradiol on the Clindamycin Sensitivity of L. gasseri and C. acnes Biofilms

#### 3.4.1. Biofilm Growth Under Standard Estradiol Administration

##### Mono-Species Biofilms

Working concentrations were set at 2.2 × 10^−10^ M estradiol and 0.5 μg/mL clindamycin to assess hormonal modulation of antibiotic activity. Data were normalized to appropriate solvent controls to isolate compound-specific effects.

In *L. gasseri* ATCC 33323, clindamycin alone inhibited biofilm formation. In contrast, combined exposure to clindamycin and estradiol resulted in a marked increase in biofilm biomass (up to 233% relative to estradiol control, *p* < 0.001; Figure 5A). A comparable stimulatory effect was observed for the clindamycin–ethanol combination (up to 198% vs. ethanol control); however, greater skews reduced its statistical significance. Ethanol alone had no effect.

For *L. gasseri* MA4, clindamycin significantly inhibited biofilm formation (35% reduction vs. untreated control, *p* < 0.001; Figure 5B). This inhibitory effect persisted in the presence of ethanol and estradiol (24%, *p* < 0.001), although a modest attenuation was observed when the antibiotic was combined with estradiol (12% reduction vs. estradiol control, *p* < 0.001).

In *C. acnes* HM514, clindamycin stimulated biofilm growth both alone (up to 126% of control, *p* < 0.005) and in combination with ethanol (13.2% vs. ethanol control, *p* < 0.05; Figure 6A). Estradiol also stimulated HM514. However, estradiol significantly reduced clindamycin activity (11%, *p* < 0.005, Figure 6A).

In contrast, *C. acnes* EAB1 displayed pronounced hormonal modulation. Clindamycin inhibited biofilm growth (20% of control, *p* < 0.05; Figure 6B). In combination with ethanol, it had a greater effect (51%, *p* < 0.001). Notably, the addition of estradiol attenuated this inhibitory effect (23%, *p* < 0.2), making the sample vary despite the inhibition tendency.

##### Effect of Estradiol on the Clindamycin Sensitivity of Microorganisms in Dual-Species Biofilms

Following mono-species assays, dual-species biofilms were established by co-culturing each *L. gasseri* strain with each *C. acnes* isolate.

**Figure 7:**
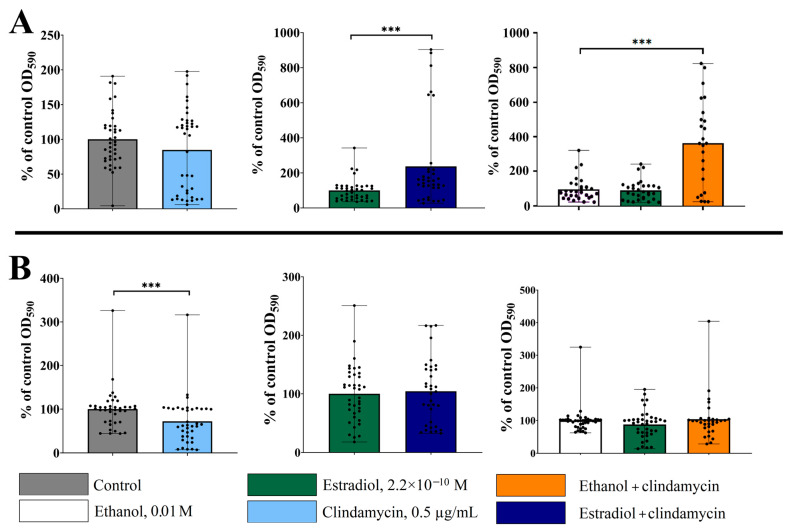
Effects of estradiol (2.2 × 10^−10^ M), clindamycin (0.5 μg/mL), and their combination on the growth of dual-species biofilms. (**A**)—*L. gasseri* ATCC 33323 and *C. acnes* HM514; (**B**)—*L. gasseri* ATCC 33323 and *C. acnes* EAB1. *** denotes *p* < 0.005. Statistical analysis was performed using the Mann–Whitney U test.

**Figure 8:**
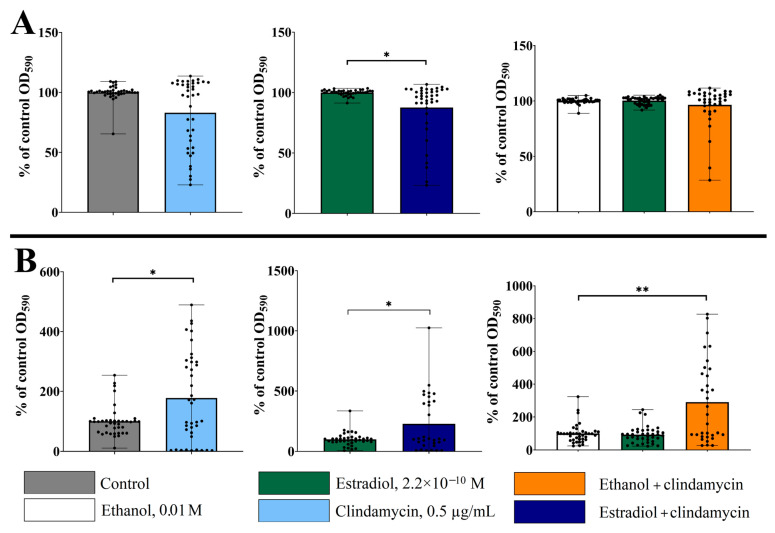
Effects of estradiol (2.2 × 10^−10^ M), clindamycin (0.5 μg/mL), and their combination on the growth of dual-species biofilms. (**A**)—*L. gasseri* MA4 and *C. acnes* HM514; (**B**)—*L. gasseri* MA4 and *C. acnes* EAB1. * denotes *p* < 0.05; ** denotes *p* < 0.01. Statistical analysis was performed using the Mann–Whitney U test.

In the *L. gasseri* ATCC 33323—*C. acnes* HM514 community, clindamycin (0.5 μg/mL) showed weak, non-significant inhibition (Figure 7A). In contrast, the clindamycin–ethanol combination markedly stimulated biofilm formation (up to 364%, *p* < 0.005). This stimulatory effect persisted in the presence of estradiol, although at reduced magnitude (237% relative to estradiol control, *p* < 0.005; Figure 8A).

For the *L. gasseri* ATCC 33323—*C. acnes* EAB1 community, clindamycin significantly inhibited biofilm growth (29% reduction, *p* < 0.005; Figure 7B). However, this inhibitory effect was abolished in the presence of ethanol. Addition of estradiol did not restore antibiotic activity, with biofilm levels remaining comparable to estradiol-treated controls.

In the *L. gasseri* MA4—*C. acnes* HM514 community, clindamycin inhibited biofilm growth (to 75.5% of control, *p* < 0.2; Figure 8A), consistent with the response of *L. gasseri* MA4 in mono-species culture. This inhibition was completely neutralized when clindamycin was combined with ethanol, while estradiol produced only a minor, non-significant effect (96.4% of ethanol control).

Unexpectedly, in the *L. gasseri* MA4—*C. acnes* EAB1 community, clindamycin induced biofilm stimulation (178%, *p* < 0.05) despite both strains being individually sensitive to the antibiotic. This effect persisted in the presence of ethanol and estradiol (respectively: 290%, *p* < 0.01 and 226%, *p* < 0.05; Figure 8B).

#### 3.4.2. Effect of Estradiol Pre-Adsorption from Different Solvents on Biofilm Growth

Based on prior evidence that surface-adsorbed estradiol can exert biological activity, we compared the effects of pre-adsorbed estradiol on clindamycin susceptibility with those obtained using standard hormone inoculation.

**Figure 9:**
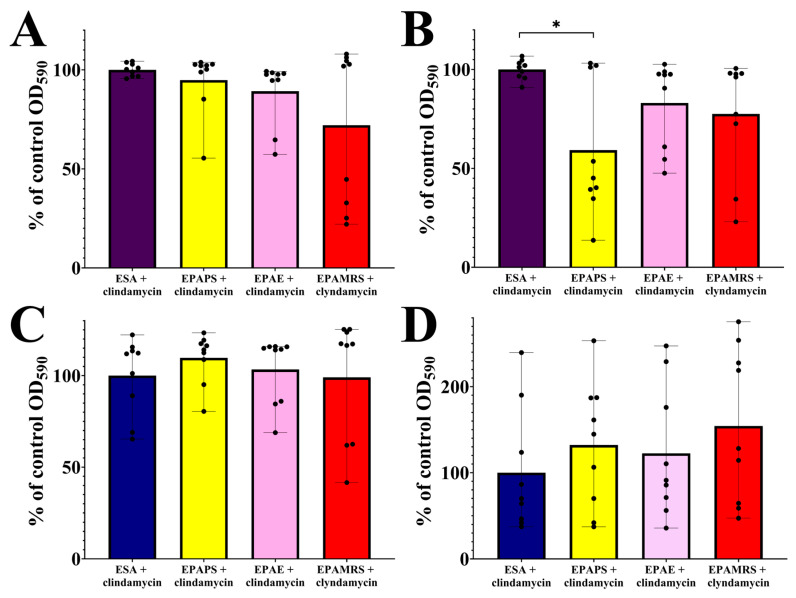
Comparative analysis of the modulatory effects of estradiol on the clindamycin susceptibility of mono-species and dual-species biofilms of *L. gasseri* and *C. acnes*. The effects of estradiol introduced via standard inoculation are compared with those of estradiol pre-adsorbed from saline, ethanol, or sterile MRS medium. (**A**)—Mono-species biofilms of *L. gasseri* ATCC 33323; (**B**)—Mono-species biofilms of *L. gasseri* MA4; (**C**)—Mono-species biofilms of *C. acnes* HM514; (**D**)—Mono-species biofilms of *C. acnes* EAB1; ESA: Estradiol Standard Administration; EPAPS: Estradiol Pre-Adsorption from Physiological Saline; EPAE: Estradiol Pre-Adsorption from Ethanol; EPAMRS: Estradiol Pre-Adsorption from MRS medium. * denotes *p* < 0.05. Statistical analysis was performed using the Mann–Whitney U test.

In mono-species biofilms, estradiol pre-adsorption did not significantly alter clindamycin sensitivity relative to standard administration across all strains (Figure 9). Only *L. gasseri* MA4 showed a shift in response under pre-adsorbed conditions: in EPAPS samples, the inhibitory effect of clindamycin returned (59%, *p* < 0.05; Figure 9B).

**Figure 10:**
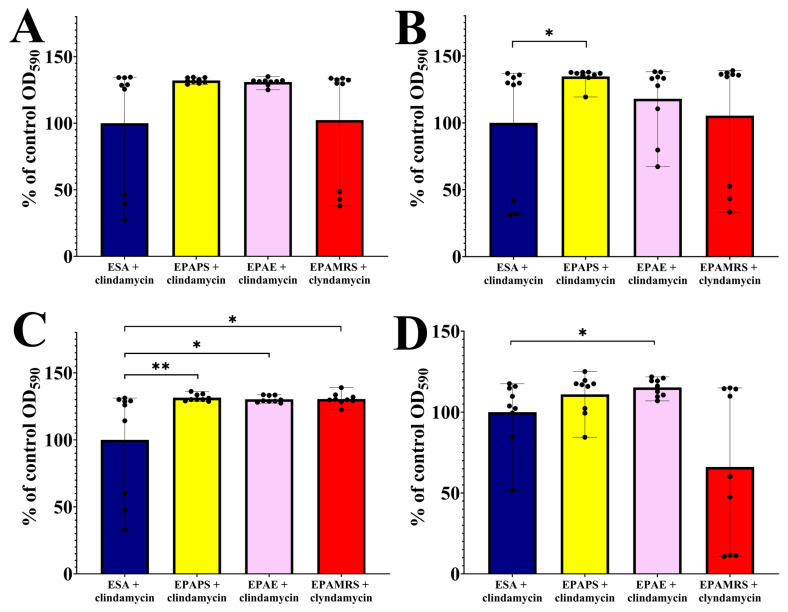
Comparative analysis of the modulatory effects of estradiol on the clindamycin susceptibility of dual-species biofilms of *L. gasseri* and *C. acnes*. (**A**)—Dual-species community of *L. gasseri* ATCC 33323 and *C. acnes* HM514; (**B**)—Dual-species community of *L. gasseri* ATCC 33323 and *C. acnes* EAB1; (**C**)—Dual-species community of *L. gasseri* MA4 and *C. acnes* HM514; (**D**)—Dual-species community of *L. gasseri* MA4 and *C. acnes* EAB1. ESA: Estradiol Standard Administration; EPAPS: Estradiol Pre-Adsorption from Physiological Saline; EPAE: Estradiol Pre-Adsorption from Ethanol; EPAMRS: Estradiol Pre-Adsorption from MRS medium. * denotes *p* < 0.05; ** denotes *p* < 0.01;. Statistical analysis was performed using the Mann–Whitney U test.

In contrast, dual-species biofilms exhibited more frequent, though generally modest, differences. In the *L. gasseri* ATCC 33323—*C. acnes* EAB community, clindamycin induced biofilm stimulation in all sample types, and the significance was in EPAPS samples (134% increase, *p* < 0.05; Figure 10B). A comparable stimulatory trend was observed in the community *L. gasseri* MA4—*C. acnes* EAB1, the most pronounced effect was in EPAE-clindamycin samples (115%, *p* < 0.05). In contrast, in EPAMRS-clindamycin samples, there was a tendency to inhibition.

The most pronounced effects were observed in the community *L. gasseri* MA4—*C. acnes* HM514. Here, in EPAPS, EPAE and EPAMRS + clindamycin samples, there was about 30% stimulation (*p* < 0.01 or *p* < 0.05; Figure 10C).

### 3.5. Quantification of Biofilm Viability via CFU Counting and Metabolic Activity Assay

#### 3.5.1. Assessment of Colony-Forming Unit (CFU) Amount in Biofilms

Lactobacilli exhibited more pronounced growth on MRS, whereas cutibacteria grew preferentially on RCM. When enumerating colony-forming units in mixed cultures, differences in colony morphology served as an additional distinguishing criterion: cutibacterial colonies were characterized by a pinkish coloration. The results were further verified by light microscopy, which was employed to examine cell morphology from each colony variant.

**Figure 11:**
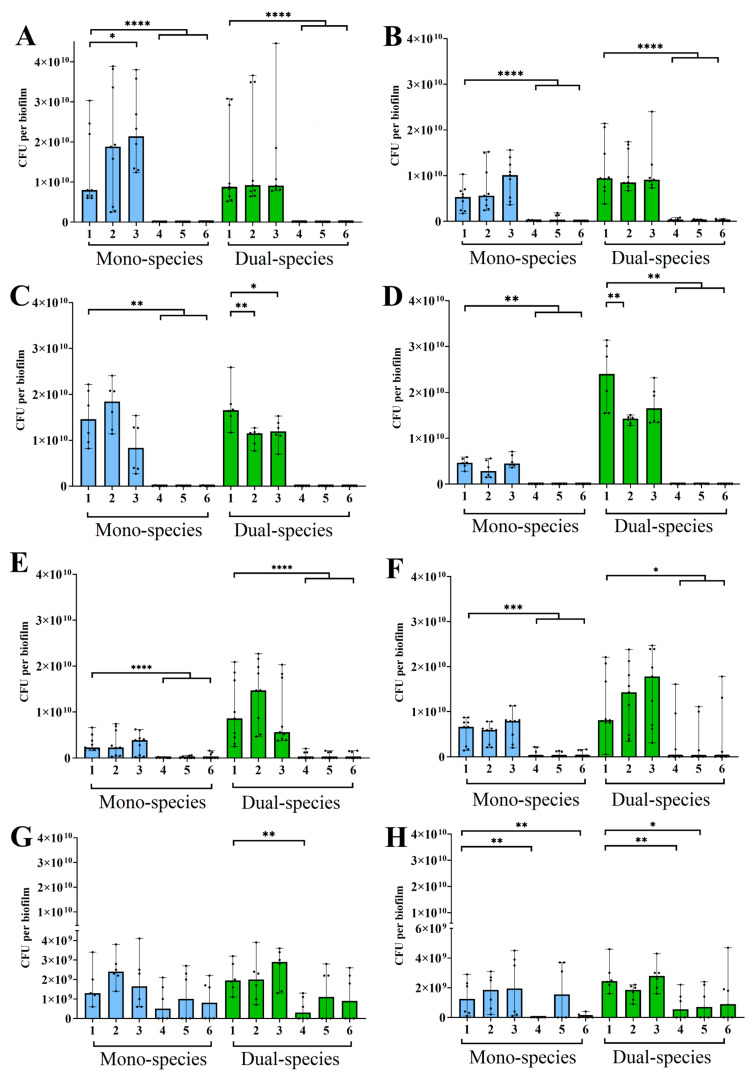
CFU counts of *L. gasseri* and *C. acnes* in mono- and dual-species biofilms. (**A**)—CFU counts of *L. gasseri* ATCC 33323 in mono-species biofilms and in community with *C. acnes* HM514; (**B**)—CFU counts of *C. acnes* HM514 in mono-species biofilms and in community with *L. gasseri* ATCC 33323; (**C**)—CFU counts of *L. gasseri* ATCC 33323 in mono-species biofilms and in community with *C. acnes* EAB1; (**D**)—CFU counts of *C. acnes* EAB1 in mono-species biofilms and in community with *L. gasseri* ATCC 33323; (**E**)—CFU counts of *L. gasseri* MA4 in mono-species biofilms and in community with *C. acnes* HM514; (**F**)—CFU counts of *C. acnes* HM514 in mono-species biofilms and in community with *L. gasseri* MA4; (**G**)—CFU counts of *L. gasseri* MA4 in mono-species biofilms and in community with *C. acnes* EAB1; (**H**)—CFU counts of *C. acnes* EAB1 in mono-species biofilms and in community with *L. gasseri* MA4. 1—control; 2—ethanol (0.01 M); 3—estradiol (2.2 × 10^−10^ M); 4—clindamycin (0.5 µg/mL); 5—ethanol (0.01 M) and clindamycin (0.5 µg/mL); 6—estradiol (2.2 × 10^−10^ M) and clindamycin (0.5 µg/mL). * denotes *p* < 0.05; ** denotes *p* < 0.01; *** denotes *p* < 0.005; **** denotes *p* < 0.001. Statistical analysis was performed using the Mann–Whitney U test.

In mono-species *L. gasseri* ATCC 33323 biofilms, estradiol produced a moderate, non-significant increase in CFU counts (Figure 11A). This effect was not observed in dual-species biofilms with *C. acnes* HM514, where lactobacilli counts remained unchanged. In mono-species *C. acnes* HM514 biofilms, combined exposure to estradiol and clindamycin resulted in a modest, non-significant reduction in viability (Figure 11B).

Distinct dynamics were observed in communities containing the vaginal isolate *C. acnes* EAB1. In dual-species biofilms with *L. gasseri* ATCC 33323, clindamycin treatment completely abolished detectable lactobacilli CFU (*p* < 0.001; Figure 11C), while ethanol alone significantly reduced *L. gasseri* viability. In contrast, under control conditions, both species exhibited enhanced growth in dual-species biofilms relative to mono-species cultures, indicating mutualistic interactions (Figure 11D).

In biofilms containing *L. gasseri* MA4, clindamycin caused pronounced reductions in CFU counts in both mono- and dual-species cultures (*p* < 0.001; Figure 11E,G). Estradiol slightly attenuated this inhibition, although differences did not reach statistical significance. In dual-species biofilms with *C. acnes* HM514, clindamycin sensitivity of *L. gasseri* MA4 was increased, while combined estradiol–clindamycin exposure partially restored lactobacilli viability.

Notably, *L. gasseri* MA4 exerted a protective effect on *C. acnes* EAB1 in dual-species biofilms, as cutibacteria remained cultivable under clindamycin exposure (Figure 11H). This effect was not observed in communities containing *L. gasseri* ATCC 33323.

#### 3.5.2. Metabolic Activity and Viability Within Dual-Species Biofilms

Analysis of overall metabolic activity (MTT assay) revealed that dual-species communities were largely dominated by the metabolic profiles of the *C. acnes* components, particularly in groups involving the HM514 strain. In most cases, clindamycin and estradiol exposure did not result in statistically significant shifts in total metabolic activity, even when corresponding mono-species biofilms showed sensitivity. This lack of metabolic response, despite changes in other parameters, suggests that community-level metabolism remains resilient or is governed by the dominant species within the biofilm niche. Detailed metabolic profiles and corresponding data for all tested combinations are provided in Supplementary Materials Section S3 and Figure S3.

### 3.6. Antibacterial Properties of L. gasseri and C. acnes

**Figure 12:**
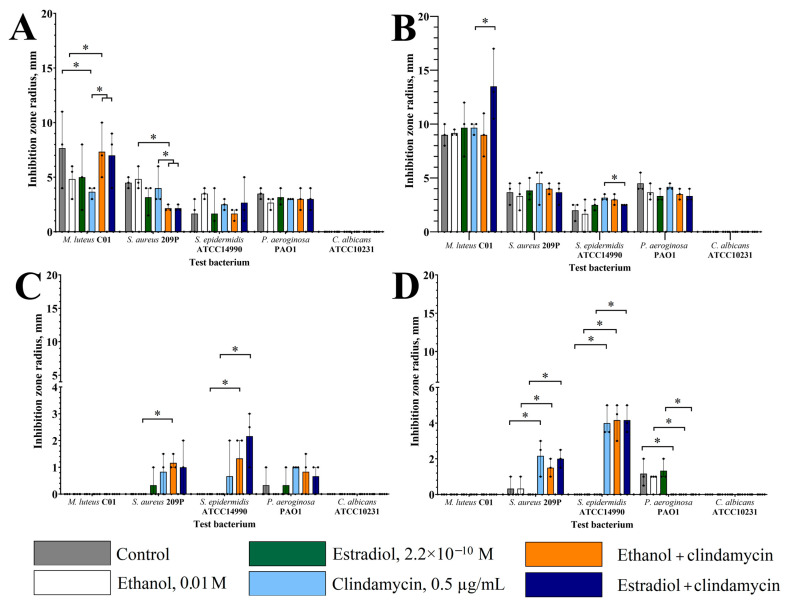
Antimicrobial activity of *L. gasseri* and *C. acnes* strains against Gram-positive and Gram-negative test bacteria and the yeast *C. albicans*. (**A**)—*L. gasseri* ATCC 33323; (**B**)—*L. gasseri* MA4; (**C**)—*C. acnes* HM514; (**D**)—*C. acnes* EAB1. * denotes *p* < 0.05. Statistical analysis was performed using the Mann–Whitney U test.

Both *L. gasseri* strains exhibited their strongest antagonistic activity against *M. luteus* C01, with *L. gasseri* ATCC 33323 producing inhibition zones with a mean radius of 7.7 mm under control conditions (Figure 12A). In this strain, the addition of clindamycin resulted in a more than two-fold reduction in antibacterial activity against *M. luteus* (3.7 mm, *p* < 0.05), whereas this effect was not observed for other test microorganisms or for the MA4 strain. Co-administration of clindamycin with either ethanol or estradiol restored antagonistic activity against *M. luteus* to near-control levels in ATCC 33323. A similar restorative effect of the clindamycin–estradiol combination was observed for *L. gasseri* MA4, where inhibition zones increased significantly compared to clindamycin alone (Figure 12B).

In contrast, the combination of clindamycin with ethanol or estradiol reduced the antagonistic activity of *L. gasseri* against staphylococci. For ATCC 33323, inhibition of *S. aureus* 209P decreased significantly under combined treatments, and a comparable reduction was observed for *L. gasseri* MA4 against *S. epidermidis* ATCC 14990. Antibacterial activity of both lactobacilli strains against *P. aeruginosa* PAO1 was low and was not significantly altered by any of the tested conditions. Neither strain exhibited inhibitory activity against *C. albicans* ATCC 10231 under any condition.

Despite the absence of cutimycin-associated genes, both *C. acnes* strains demonstrated inhibitory activity against *S. aureus* 209P, *S. epidermidis* ATCC 14990, and *P. aeruginosa* PAO1, whereas *M. luteus* C01 was resistant to *C. acnes*-mediated inhibition. The addition of clindamycin enhanced the antagonistic activity of both *C. acnes* strains against staphylococci, with detectable inhibition in all clindamycin-containing samples. In strain HM514, inhibition zones against *S. epidermidis* increased further when clindamycin was combined with ethanol or estradiol, whereas strain EAB1 exhibited relatively uniform inhibition across clindamycin-containing conditions (Figure 12C). Responses to *P. aeruginosa* differed between strains: HM514 retained inhibitory activity under clindamycin exposure, while EAB1 lost detectable anti-pseudomonal activity in the presence of the antibiotic (Figure 12D). Similar to lactobacilli, neither *C. acnes* strain inhibited the growth of *C. albicans* under any tested condition.

The bacterial strains studied also displayed a degree of mutual antagonism, which was observed even at the intra-species (strain) level. For a detailed account, see Supplementary Materials Section S4.

### 3.7. Study of Differential Bacteriocin Gene Expression in L. gasseri and C. acnes

#### Detection and Expression of Gassericin Genes in *L. gasseri*

PCR analysis confirmed the presence of all target gassericin-encoding genes in both *L. gasseri* strains. Due to biofilm-associated heterogeneity, only expression changes that were consistent across all three biological replicates relative to the untreated control were considered. We assessed the expression of 16S rRNA gene stability across several representative conditions and observed no significant variation.

**Figure 13:**
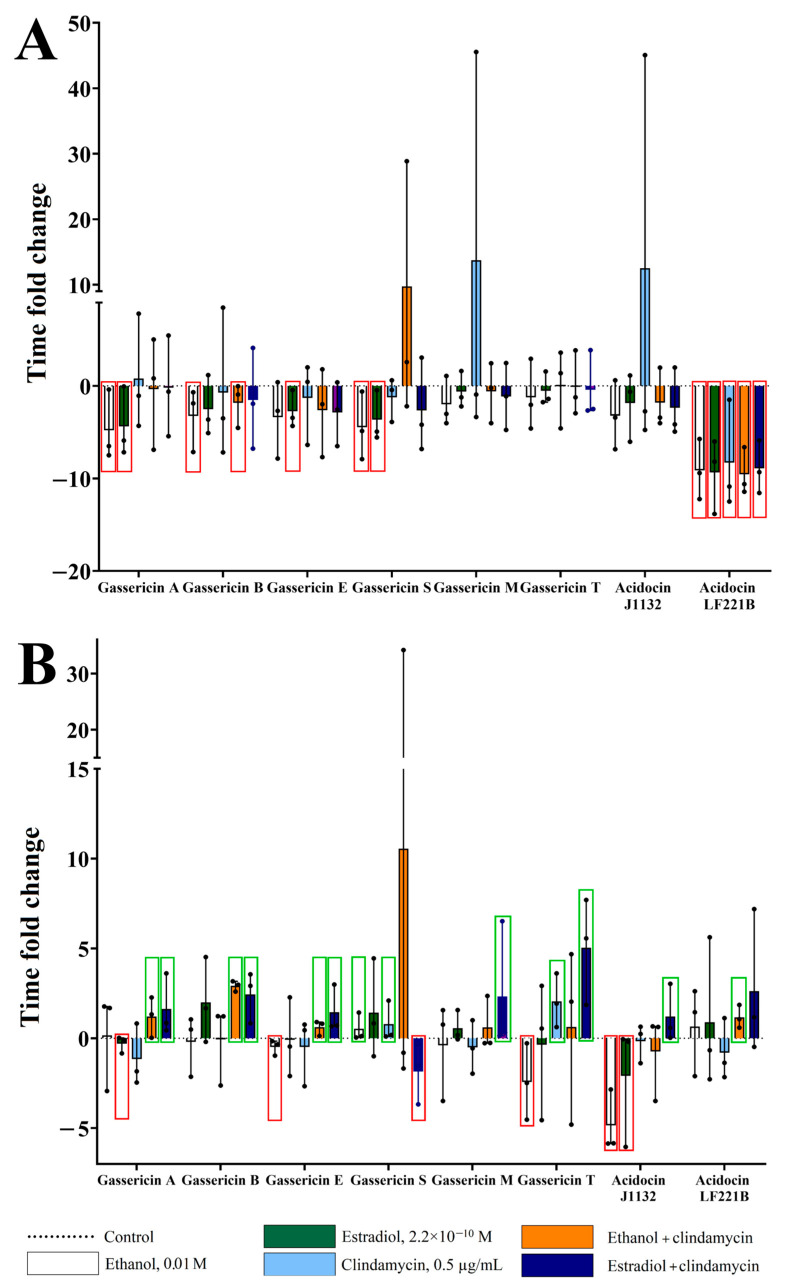
Differential expression of gassericin-encoding genes in *L. gasseri* strains. (**A**)—*L. gasseri* ATCC 33323; (**B**)—*L. gasseri* MA4. Red frames indicate consistent downregulation of gene expression; green frames indicate consistent upregulation of gene expression. Statistical analysis was performed using the Mann–Whitney U test.

In *L. gasseri* ATCC 33323, exposure to active compounds predominantly resulted in suppression of bacteriocin gene expression. Expression of gassericins A, B, E, and S was consistently downregulated in the presence of ethanol, estradiol, and the ethanol–clindamycin combination. In addition, acidocin LF221B expression was reduced under all tested conditions (Figure 13A).

In contrast, *L. gasseri* MA4 exhibited a distinct transcriptional response. Ethanol and estradiol alone generally suppressed bacteriocin gene expression, with the exception of gassericin S, which was upregulated (Figure 13B). Conversely, combinations of clindamycin with either ethanol or estradiol resulted in consistent upregulation of gassericins A, B, E, M, T, and acidocin genes, while the clindamycin–estradiol combination led to suppression of gassericin S expression.

**Figure 14:**
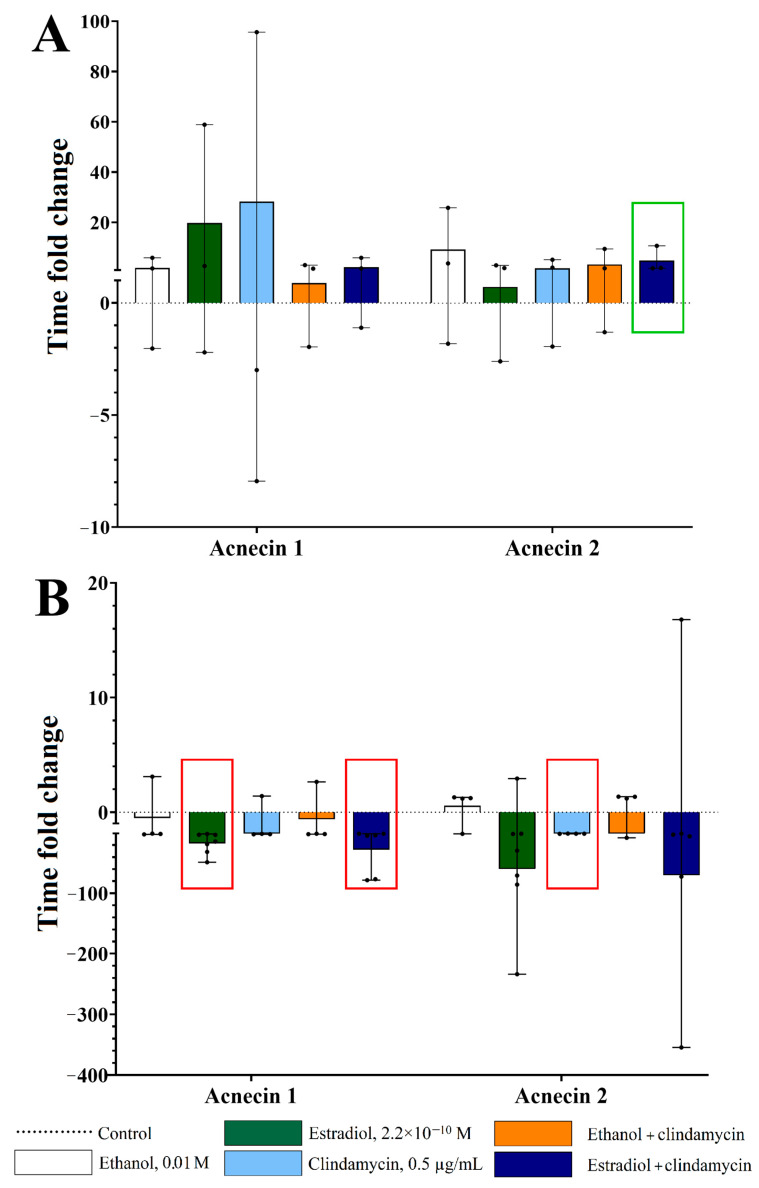
Differential expression of acnecin-encoding genes in *C. acnes* strains. (**A**)—*C. acnes* HM514; (**B**)—*C. acnes* EAB1. Red frames indicate consistent downregulation of gene expression; green frames indicate consistent upregulation of gene expression. Statistical analysis was performed using the Mann–Whitney U test.

In *C. acnes*, transcriptional responses to active compounds showed pronounced strain specificity (Figure 14). In strain HM514, active compounds tended to stimulate gene expression, although most changes were inconsistent across replicates. Stable upregulation was detected only for the acnecin II gene in the presence of the estradiol–clindamycin combination (Figure 14A). In contrast, strain EAB1 displayed a general trend toward downregulation, with consistent suppression of acnecin I expression following estradiol or estradiol–clindamycin treatment and downregulation of acnecin II in the presence of clindamycin alone (Figure 14B).

Despite these transcriptional changes, no consistent association was observed between acnecin gene expression patterns and antibacterial activity in either *C. acnes* strain. Intraspecific antagonistic activity remained unchanged across conditions, including those associated with altered acnecin transcription (Supplementary Materials Section S4, Figure S4C,D).

## 4. Discussion

This pilot study demonstrates that estradiol, clindamycin, and ethanol exert complex, strain-dependent effects on *L. gasseri* and *C. acnes*. Our findings underscore a multifactorial regulatory landscape where hormonal and antibiotic impacts are governed by strain identity, community composition, and surface context, aligning with previous reports on high intra-species variability [47].

In mono-species biofilms, estradiol showed pronounced strain-specificity, inhibiting *L. gasseri* at physiological levels while displaying divergent effects on *C. acnes* (stimulating the skin-derived HM514 but inhibiting the vaginal EAB1). Notably, growth kinetic changes did not always match biomass accumulation; for instance, in *L. gasseri* ATCC 33323, estradiol attenuated growth rates without reducing final biomass, whereas *L. gasseri* MA4 displayed a biphasic response, with mild stimulation at physiological concentrations and inhibition at higher doses. Such discrepancies highlight the importance of distinguishing between proliferation kinetics and adherent biomass quantification.

Throughout our experimental systems, clindamycin was applied at sub-inhibitory concentrations. From a clinical perspective, such levels represent a potential scenario often resulting from suboptimal dosing, poor patient compliance, or fluctuating drug bioavailability within the vaginal niche. Our results reinforced strain-specificity: while strongly inhibiting *L. gasseri*, clindamycin provided less reduction in *C. acnes* EAB1 or even stimulated biofilm growth in *C. acnes* HM514. When combined with the antibiotic, estradiol often acted as a non-additive modulator, attenuating clindamycin’s potency in both *L. gasseri* and the vaginal *C. acnes* EAB1. This suggests that hormonal backgrounds can directly mitigate antibiotic efficacy, especially when drug concentrations fall below therapeutic thresholds. Further, pronounced strain-specific differences observed between the vaginal isolate (*C. acnes* EAB1) and the skin-derived isolate (*C. acnes* HM514) under estradiol exposure may reflect niche-associated functional adaptations within the species. In this context, it is plausible that the vaginal isolate possesses distinct genomic or regulatory features that confer altered responsiveness to hormonal stimuli, particularly in pathways related to membrane composition, nutrient utilization, and cell surface interactions. Such adaptations may underlie the differential physiological responses observed between the two isolates in the present study.

Community context further reshaped these interactions. In dual-species biofilms, antibiotic effects observed in mono-species systems were frequently altered or even reversed. For example, clindamycin stimulated biofilm growth in *L. gasseri* MA4—*C. acnes* EAB1 communities despite inhibiting both species individually. Potentially, such an effect may occur due to a general increase in biomass growth and decrease in its stability, which results in bigger skews and data points dispersal. Furthermore, the key factors underlying this alteration in the active substance’s effect are likely the interactions among microorganisms within the community and the resulting change in the composition of the extracellular polymeric matrix, which warrants further investigation. In contrast, in *L. gasseri* ATCC33323—*C. acnes* EAB1 pair showed weak inhibition of mono-species biofilms, which transformed into suppression within the dual-species community. These findings indicate that antibiotic efficacy cannot be reliably inferred from mono-species assays alone and is instead governed by competitive interactions, dominance effects, and stress redistribution within microbial communities. The observed dynamics in dual-species biofilms serve as further evidence of the inherent complexity and unpredictability of even the simplest microbial communities. In these systems, mono-species trends were frequently reversed, with clindamycin stimulating mixed communities despite inhibiting each species individually. This confirms that antibiotic efficacy is governed by competitive dominance and stress redistribution rather than intrinsic susceptibility alone.

Furthermore, the fact that estradiol exerts its regulatory influence both when dissolved in the medium and when pre-adsorbed onto the surface is consistent with the hypothesis of a mechanism involving receptor-like surface structures in Gram-positive bacteria [26], although direct evidence for such structures remains lacking. As an alternative, the hormone could trigger a membrane stress response, as previously demonstrated in *P. aeruginosa* [48], suggesting that estradiol may act by modulating membrane integrity or signaling pathways associated with surface sensing. Pre-adsorption of the hormone onto polystyrene surfaces tended to enhance its biological activity compared to standard administration, particularly in dual-species biofilms, although the magnitude of these effects was often modest and varied. Notably, the finding that estradiol was more effective in a pre-adsorbed state aligns with the surface-associated hypothesis and raises the possibility that its regulatory impacts are mediated, at least in part, through localized interactions at the cell–substrate interface. Adsorption from physiological saline produced the most stable effects, whereas MRS- or ethanol-based pre-adsorption increased the variability of the outcomes. Although in some cases the statistically significant differences were modest, the recurrence of this trend lends preliminary support to the idea that surface-bound estradiol may influence early adhesion and biofilm architecture in line with previous observations obtained in eukaryotic models [49]. These data are suggestive of a direct signaling role for estradiol, but they do not exclude indirect mechanisms. Moreover, surface-bound estradiol generally showed enhanced activity, particularly in mixed-species biofilms, suggesting that these interactions are crucial for early adhesion and architecture. Finally, polystyrene is known to accumulate estradiol [50], which could increase its local bioavailability at the interface between bacteria and plates. However, little is known about changes in local hydrophobicity caused by hormone accumulation. It is plausible that alterations in the local microenvironment could partly account for the observed results. It is also known that the concentration of estradiol in vaginal tissues differs from that in blood plasma [51]. In postmenopausal women, it is typically an order of magnitude lower [52,53,54]. In younger women, the concentration varies depending on the menstrual cycle [55]. Taken together, these findings provide indirect, preliminary evidence that a potential decrease may play a role in the fact that the concentration of estradiol decreases upon its pre-adsorption onto the surface, thereby altering the effect of the hormone on biofilms, although definitive mechanistic studies will be required.

Further, we observed a distinct uncoupling between biofilm biomass (CV staining), viability (CFU counting), and metabolic activity (MTT). This discrepancy likely stems from shifts in cellular aggregation and the potential induction of a VBNC (viable but non-culturable) state under hormonal or antibiotic stress (See Supplementary Materials Section S3). Moreover, the aforementioned changes in the production of the extracellular polymeric matrix may also account for this discrepancy in the results, which requires separate investigation in the future. Such physiological heterogeneity highlights that these compounds may alter biofilm architecture and metabolic status without necessarily exerting a direct bactericidal effect, a phenomenon that warrants deeper investigation at the single-cell and molecular levels.

Similarly, transcriptional changes in bacteriocin genes did not consistently correlate with phenotypic antagonism. Given the evolutionary conservation of bacteriocin systems in *L. gasseri*, the observed transcriptional shifts may reflect a broader regulatory response of the gassericin-producing loci, although this interpretation remains tentative. While high sequence similarity between certain paralogs complicates absolute quantification; therefore, the reported gene expression data should be viewed as indicative of overall trends in gene modulation that reflect a systemic metabolic shift under hormonal and antibiotic pressure. Furthermore, gene expression patterns showed considerable variability across biological replicates, likely reflecting the high spatial and physiological heterogeneity characteristic of mature biofilms. This transcriptional noise, in conjunction with the multilayered regulation of bacteriocins, suggests that clindamycin, beyond its canonical role as a translation inhibitor, may act as a stress cue that elicits metabolic reprogramming rather than directly modulating bacteriocin peptide output. However, this hypothesis requires further experimental validation at the protein and functional levels. Finally, the agar-block diffusion assay should be clarified as measuring net diffusible antibacterial activity released from agar-grown bacterial communities, rather than total bacteriocin synthesis. Reduced or absent inhibition zones, therefore, do not necessarily indicate absence of bacteriocin production, as antimicrobial compounds may be retained or neutralised within the colony biofilm matrix, associated cells, or agar block. Moreover, bacteriocins may be susceptible to degradation by endogenous proteolytic enzymes or to general instability in the growth medium, which can reduce their detectable activity despite increased transcription of the corresponding genes.

These findings have potential clinical and microbiological relevance. Beyond the fundamental interest in how antibiotic stress reshapes microbial communities, our data highlight the risks of therapeutic failure. The apparent “inversion” of clindamycin activity in dual-species systems suggests that suboptimal treatments may fail to disrupt complex biofilms and could, under certain conditions, paradoxically promote their resilience. Our data suggest that antibiotic efficacy in this experimental system is influenced by the hormonal environment and community structure. Recognizing the impact of sub-inhibitory concentrations, whether arising from clinical errors, pharmacokinetic variability, or biofilm-related gradients, is essential for improving the predictability of antimicrobial interventions and understanding the resilience of the polymicrobial biofilms of human commensal bacteria.

## 5. Conclusions

In summary, this pilot study demonstrates that the impact of estradiol and clindamycin on lactobacilli and cutibacteria is highly contingent on strain-specific physiology. While estradiol inhibited biofilm formation in *L. gasseri* ATCC 33323 and MA4, it exerted divergent effects on the two *C. acnes* isolates tested, stimulating the skin-derived HM514 but suppressing the vaginal isolate EAB1. The apparent inversion of clindamycin activity within *L. gasseri* MA4—*C. acnes* EAB1 dual-species biofilms further underscore the limitations of traditional mono-species assays in predicting polymicrobial outcomes. Furthermore, the tendency of surface-bound estradiol to enhance biological activity is consistent with the hypothesis of a direct hormonal interaction with the bacterial cell envelope, although this interpretation requires further experimental validation. These findings highlight that improving antimicrobial strategies requires a multifactorial approach that accounts for hormonal background, community context, and the substantial phenotypic variability among individual isolates.

## Data Availability

The original contributions presented in this study are included in the article/Supplementary Materials. Further inquiries can be directed to the corresponding authors.

## Supplementary Materials

The following supporting information can be downloaded at https://www.mdpi.com/article/10.3390/microorganisms14061173/s1.
