# Supplementary material for: Estradiol-Induced Modulation of Clindamycin Susceptibility in Mono- and Dual-Species Biofilms of Lactobacillus gasseri and Cutibacterium acnes: An In Vitro Model Study
Source: Microorganisms. 2026 May 22;14(6):1173. doi: 10.3390/microorganisms14061173 (PMC13302852; doi:10.3390/microorganisms14061173)
Supplement: Supplementary file 1 [file microorganisms-14-01173-s001.zip › Supplementary material S1 R1.pdf]

Table S1. Primers used in the study.

GenBank ID	Product	Primer Type	5′-3′-Sequence
L. gasseri			
KF724910.1	gassericin A	forward	GTGTTGCAGGATCATGTGGT
		reverse	TCCGTTAGCGCACCCTAATA
AY307382.1	gassericin K7B	forward	GGTGGAAACAAGTGGGGGAA
		reverse	TCCGAATCCTCTGCACCAAC
KR080485.1	gassericin E	forward	TAGGTGGAGTAGGTGGAGCG
		reverse	CCAACAAAGCCACAAGCAGG
LC389591.1	gassericin S	forward	AGCAGGAGGAGCATTTTCAA
		reverse	CCTGCTGCACCACCTAAAAT
AB710328.1	gassericin T	forward	TGGAAATGCAGTTTGCGGTC
		reverse	TTACGCCAGCCCATGCTATT
AY295874.1	acidocin LF221A	forward	ACATGCTGCTGAGCCTTTGA
		reverse	GTCAGGCAGTTGGTGCATTG
AY297947.1	Acidocin LF221B	forward	ATGGGTGGAAACAAGTGGGG
		reverse	CCGAATCCTCTGCACCAACT
AB517146.1	16S rRNA	forward	ACTAGATACAAGCGAGCGGC
		reverse	TTGCTCCATCAGACTTGCGT
C. acnes			
WP_002523776.1	acnecin I	forward	GTGCGAGCATAGGACCAACT
		reverse	TACGGACGTGGATACTCGGA
WP_014167175.1	acnecin II	forward	GATCTTGCAGGTCGCGGATA
		reverse	AGCCTCGACTACACAAAGCC
ON394600.1	16S rRNA	forward	GTAAACCGCTTTCGCCTGTG
		reverse	CAACCACCTACGAGCCCTTT
