# Supplementary material for: Estradiol-Induced Modulation of Clindamycin Susceptibility in Mono- and Dual-Species Biofilms of Lactobacillus gasseri and Cutibacterium acnes: An In Vitro Model Study
Source: Microorganisms. 2026 May 22;14(6):1173. doi: 10.3390/microorganisms14061173 (PMC13302852; doi:10.3390/microorganisms14061173)
Supplement: Supplementary file 1 [file microorganisms-14-01173-s001.zip › Supplementary material Section S2 R1.pdf]

## Supplementary Section S2. Detailed Analysis of Microbial Growth Kinetics under Estradiol and Clindamycin Exposure

### S2.1. Influence of Estradiol on the Biofilm Formation Kinetics

For *L. gasseri* ATCC 33323 (Fig. S1A), ethanol had no significant effect on growth kinetics. In contrast, physiological estradiol (2.2 × $10^{-10}$ M) reduced the specific growth rate by ~30% (0.20 vs 0.29 $h^{-1}$) and increased generation time (3.47 vs 2.35 h), with a minor reduction in $OD_{max}$ at higher hormone concentrations.

For *L. gasseri* MA4 (Fig. S1B), physiological estradiol slightly stimulated growth, increasing μ by ~11% and reducing generation time (3.22 vs 4.52 h), while final biomass was only marginally affected (~6% reduction). At higher estradiol concentrations, growth inhibition became evident, with $\mu$ declining to 0.139 $h^{-1}$ and generation time extending to 6.11 h, indicating a biphasic, dose-dependent response.

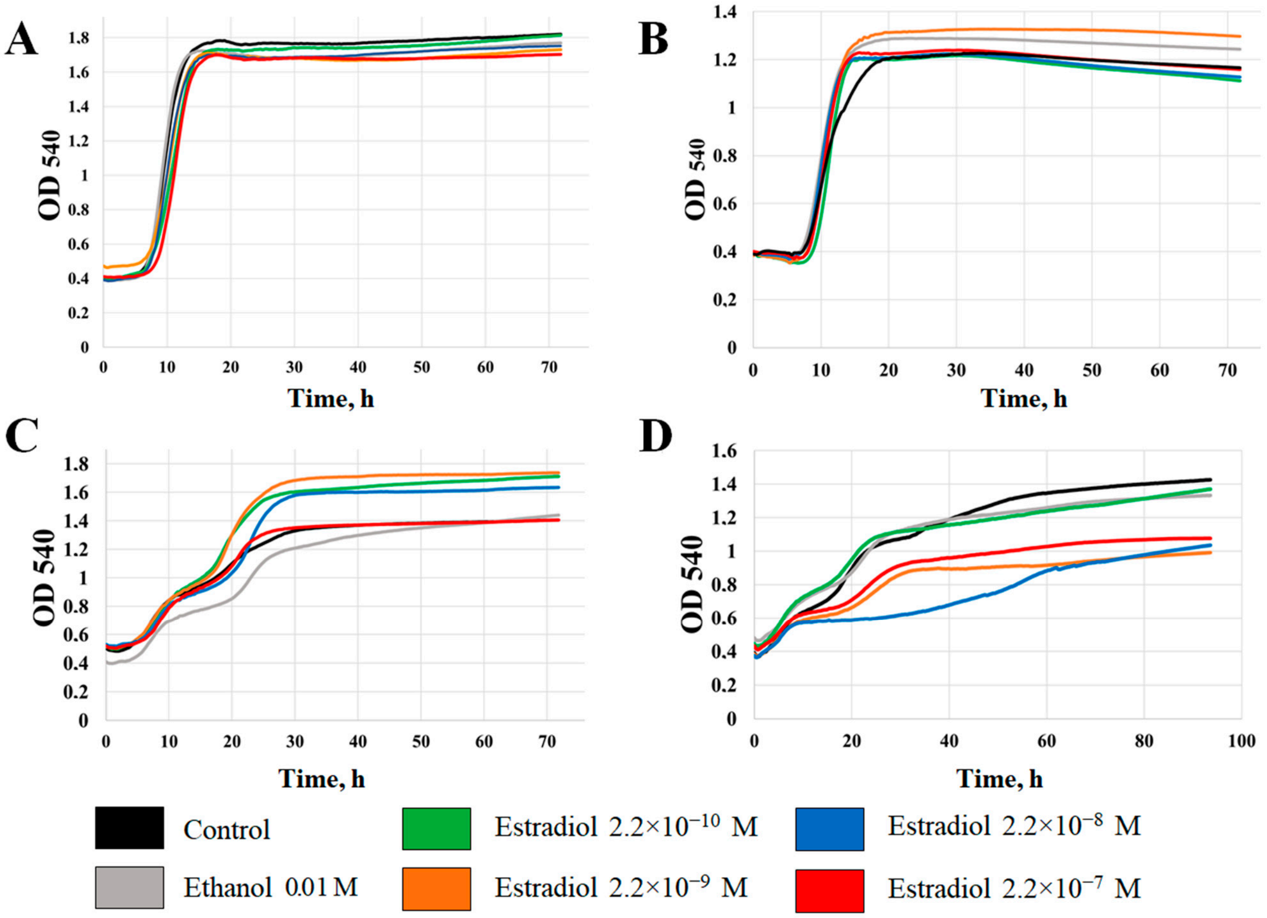


**Figure S1.** Growth curves of the studied strains under estradiol exposure. A–*L. gasseri* ATCC 33323; B–*L. gasseri* MA4; C–*C. acnes* HM514; D–*C. acnes* EAB1.

In *C. acnes* HM514 (Fig. S1C), ethanol markedly suppressed growth, reducing μ by ~45% and prolonging generation time. Estradiol antagonized this effect, partially restoring growth kinetics toward control values, with the strongest recovery observed at physiological concentrations. $OD_{max}$ also increased under estradiol exposure relative to both control and ethanol-treated cultures.

By contrast, *C. acnes* EAB1 (Fig. S1D) exhibited enhanced sensitivity to estradiol. While ethanol alone reduced $\mu$ by ~30%, increasing estradiol concentrations further exacerbated growth inhibition. At the highest hormone concentration, $\mu$ decreased by ~26%

relative to the ethanol control, and generation time nearly doubled. Physiological estradiol produced only minor kinetic changes and did not counteract ethanol-induced suppression.

## S2.2. Influence of clindamycin on the Biofilm Formation Kinetics

Both *L. gasseri* strains were highly sensitive to clindamycin. In *L. gasseri* ATCC 33323, growth inhibition was dose-dependent, beginning at 0.1 µg/mL (≈22% reduction in µ, with increased generation time) and culminating in near-complete growth arrest at 0.5 µg/mL (≈94% reduction in *µ*), accompanied by a marked decrease in final biomass ($OD_{600}$ 0.88 vs 1.77 in control; Fig. S2A).

A comparable pattern was observed for *L. gasseri* MA4, where clindamycin caused a 71% reduction in *µ* at 0.1 µg/mL and >80% inhibition at 0.5 µg/mL, with generation times exceeding 120 h, confirming pronounced susceptibility (Fig. S2B).

In contrast, *C. acnes* HM514 displayed substantially greater tolerance to clindamycin. Even at 20 µg/mL, growth was only partially suppressed, with *µ* reduced by ~39% and final biomass by ~25% relative to control (Fig. S2C).

*C. acnes* EAB1 (Fig. S2D) showed higher sensitivity than HM514. At a concentration of 0.5 µg/mL, *µ* decreased by 28.78% (from 0.051 $h^{-1}$ to 0.036 $h^{-1}$), with a significant extension of the generation time from 14.77 h to 76.97 h.

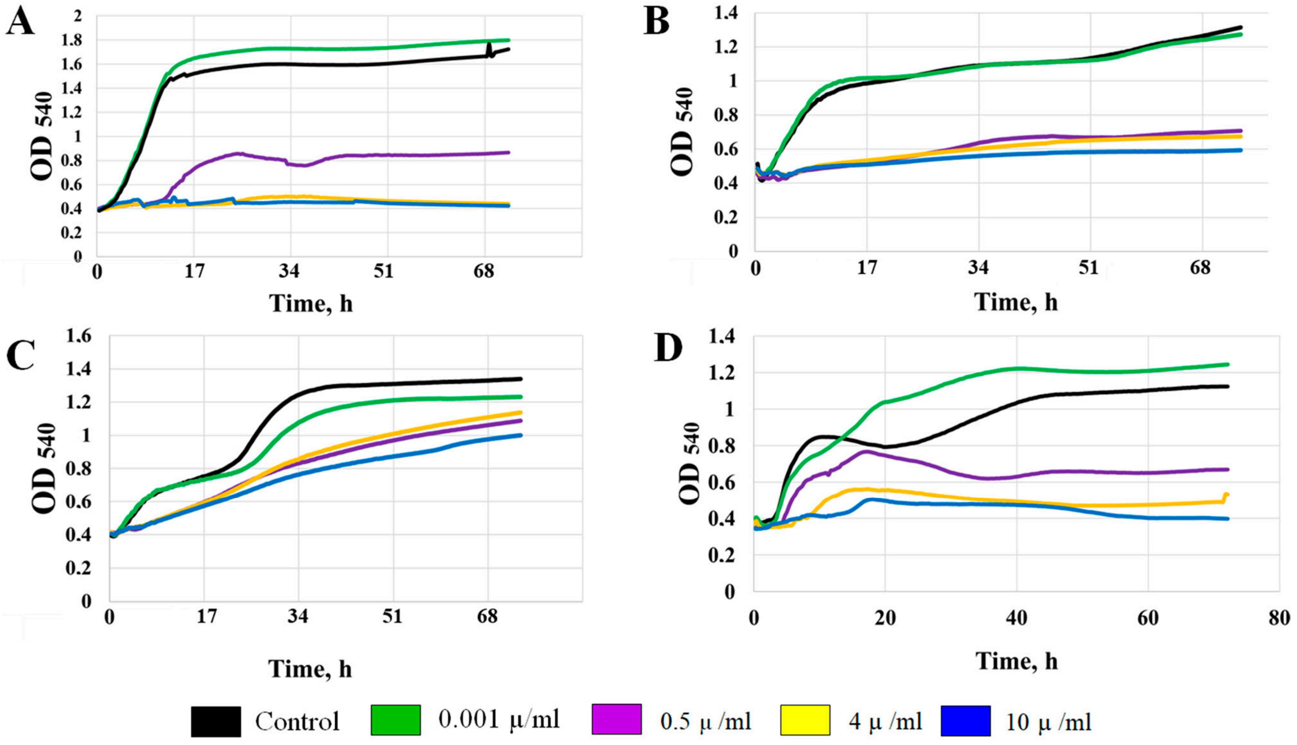


**Figure S2.** Growth curves of the studied strains under clindamycin exposure: A – *L. gasseri* ATCC 33323, B – *L. gasseri* MA4, C – *C. acnes* HM514, D – *C. acnes* EAB1 in the presence of clindamycin.
