# Supplementary material for: Estradiol-Induced Modulation of Clindamycin Susceptibility in Mono- and Dual-Species Biofilms of Lactobacillus gasseri and Cutibacterium acnes: An In Vitro Model Study
Source: Microorganisms. 2026 May 22;14(6):1173. doi: 10.3390/microorganisms14061173 (PMC13302852; doi:10.3390/microorganisms14061173)
Supplement: Supplementary file 1 [file microorganisms-14-01173-s001.zip › Supplementary material Section S3 PROOF.pdf]

Section S3. Metabolic Activity (MTT) in Mono- and Dual-Species Biofilms

In communities comprising *L. gasseri* ATCC 33323 and *C. acnes* HM514, overall metabolic activity closely resembled that of *C. acnes* HM514 mono-cultures, indicating cutibacterial dominance under competitive growth conditions (Fig. S3A). Exposure to clindamycin (0.5 µg/mL), alone or in combination with estradiol or ethanol, did not significantly alter metabolic activity in these communities, mirroring trends observed in lactobacilli mono-cultures.

In *L. gasseri* ATCC 33323 – *C. acnes* EAB1 communities, estradiol produced a minor increase in metabolic activity relative to ethanol controls; however, clindamycin-induced inhibition observed in *L. gasseri* mono-cultures (~20%) was not significantly modulated by estradiol in the dual-species community (Fig. S3B).

Similarly, in communities formed by *L. gasseri* MA4 and *C. acnes* HM514, metabolic activity patterns were again dictated by *C. acnes* HM514, with clindamycin causing only a modest reduction in activity in both mono- and dual-species settings (Fig. S3C). Estradiol showed a weak stimulatory trend in dual-species communities, although high variability prevented statistical significance.

Finally, clindamycin had no statistically significant effect on metabolic activity in *C. acnes* EAB1 mono-cultures, whereas *L. gasseri* MA4 mono-species biofilms and their corresponding dual-species communities displayed clear sensitivity to the antibiotic (Fig. S3D). In these competitive communities, estradiol did not significantly modify metabolic responses in the presence of clindamycin.

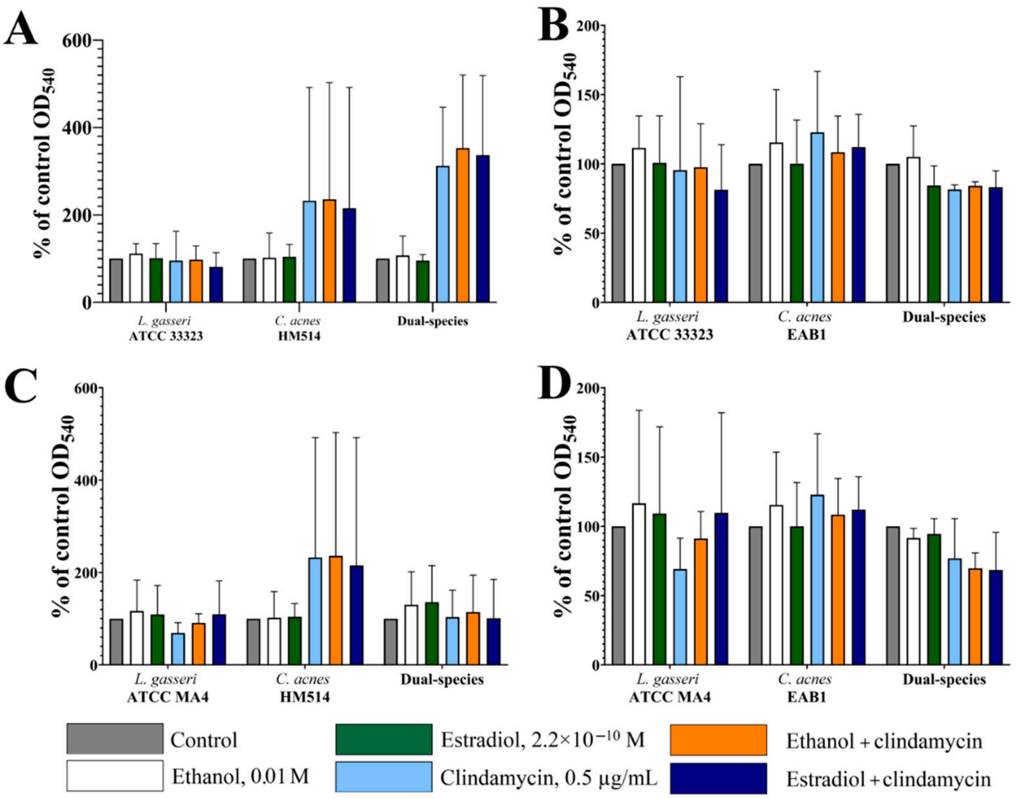

Figure S3. MTT staining of *L. gasseri* and *C. acnes* mono- and dual-species biofilms. A - monospecies and dual-species biofilms of *L. gasseri* ATCC 33323 and *C. acnes* HM514; B - monospecies and dual-species biofilms of *L. gasseri* ATCC 33323 and *C. acnes* EAB1; C - monospecies and dual-species biofilms of *L. gasseri* MA4 and *C. acnes* HM514; D - monospecies and dual-species biofilms of *L. gasseri* ATCC MA4 and *C. acnes* EAB1.
