# Supplementary material for: Estradiol-Induced Modulation of Clindamycin Susceptibility in Mono- and Dual-Species Biofilms of Lactobacillus gasseri and Cutibacterium acnes: An In Vitro Model Study
Source: Microorganisms. 2026 May 22;14(6):1173. doi: 10.3390/microorganisms14061173 (PMC13302852; doi:10.3390/microorganisms14061173)
Supplement: Supplementary file 1 [file microorganisms-14-01173-s001.zip › Supplementary Section S4 PROOF.pdf]

Supplementary Section S4. Mutual Antagonistic Activity of *L. gasseri* and *C. acnes* Strains

The presence of genes encoding cutimycin and acnecin was assessed in the studied *C. acnes* strains. PCR analysis confirmed the absence of cutimycin-related genetic markers in both HM514 and EAB1, indicating that neither strain produces this antimicrobial compound. Accordingly, baseline antibacterial activity of these strains toward heterologous species was expected to be limited.

Antagonism assays revealed pronounced strain-specific interactions (Fig. S4). *L. gasseri* MA4 exhibited the strongest inhibitory activity: a mature 72-h MA4 biofilm completely suppressed the growth of *C. acnes* HM514 and consistently inhibited *L. gasseri* ATCC 33323 (Fig. S4B). In contrast, ATCC 33323 showed only weak inhibitory activity against MA4, detectable primarily under clindamycin exposure (Fig. S4A). Both *L. gasseri* strains inhibited HM514 growth, whereas neither affected the vaginal isolate *C. acnes* EAB1.

Distinct inter-strain interactions were observed within *C. acnes*. While HM514 did not inhibit EAB1 (Fig. S4C), the vaginal isolate EAB1 significantly suppressed the growth of the skin-derived strain HM514 (Fig. S4D). Both *C. acnes* strains displayed inhibitory activity against *L. gasseri* only in the presence of clindamycin, with HM514 exerting the strongest effect. Among lactobacilli, *L. gasseri* MA4 was more susceptible to *C. acnes*-mediated inhibition than ATCC 33323.

Neither estradiol nor ethanol altered the observed antagonistic interactions, nor did they modify the clindamycin-dependent antibacterial effects.

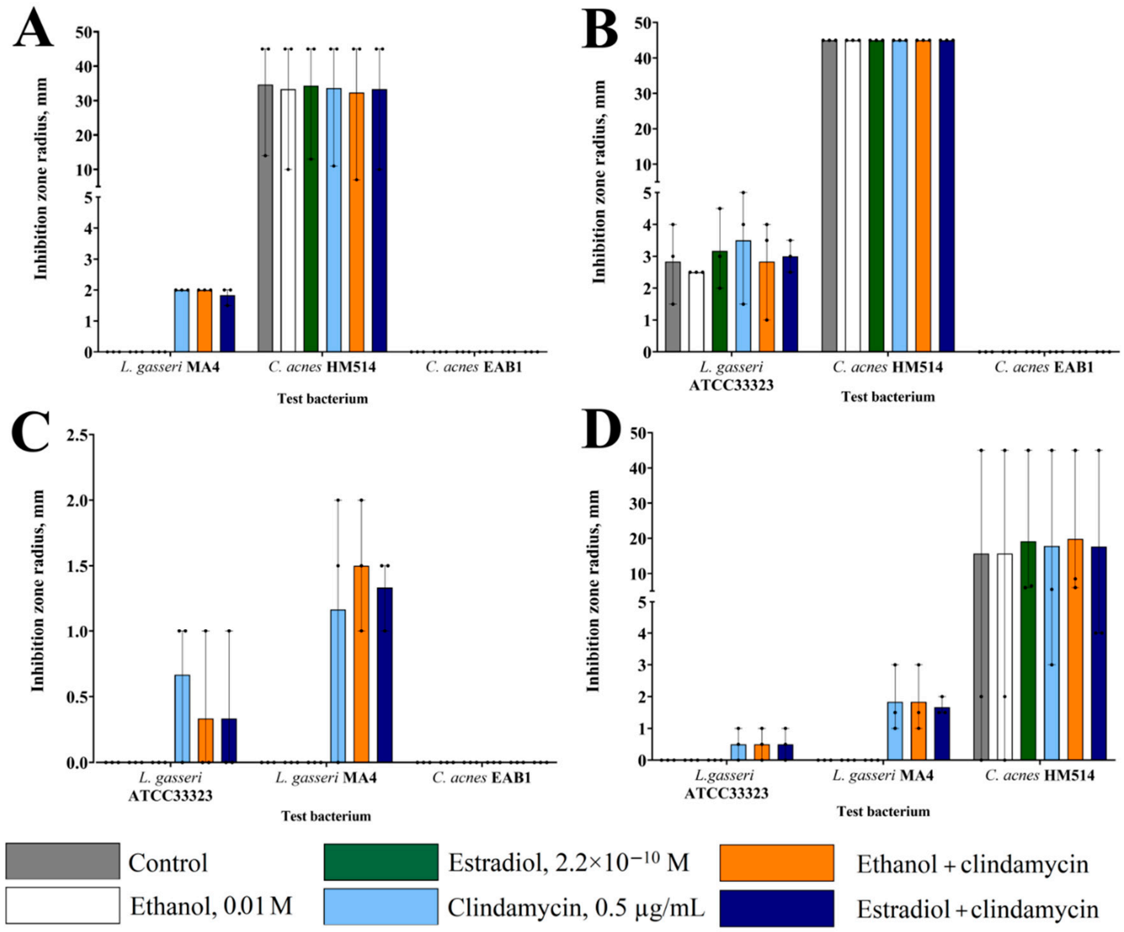

Figure S4. Mutual antibacterial activity of *L. gasseri* and *C. acnes* strains. A – *L. gasseri* ATCC 33323; B – *L. gasseri* MA4; C – *C. acnes* HM514; D – *C. acnes* EAB1.
