# Supplementary figures and images for: Estradiol-Induced Modulation of Clindamycin Susceptibility in Mono- and Dual-Species Biofilms of Lactobacillus gasseri and Cutibacterium acnes: An In Vitro Model Study
Source: Microorganisms. 2026 May 22;14(6):1173. doi: 10.3390/microorganisms14061173 (PMC13302852; doi:10.3390/microorganisms14061173)

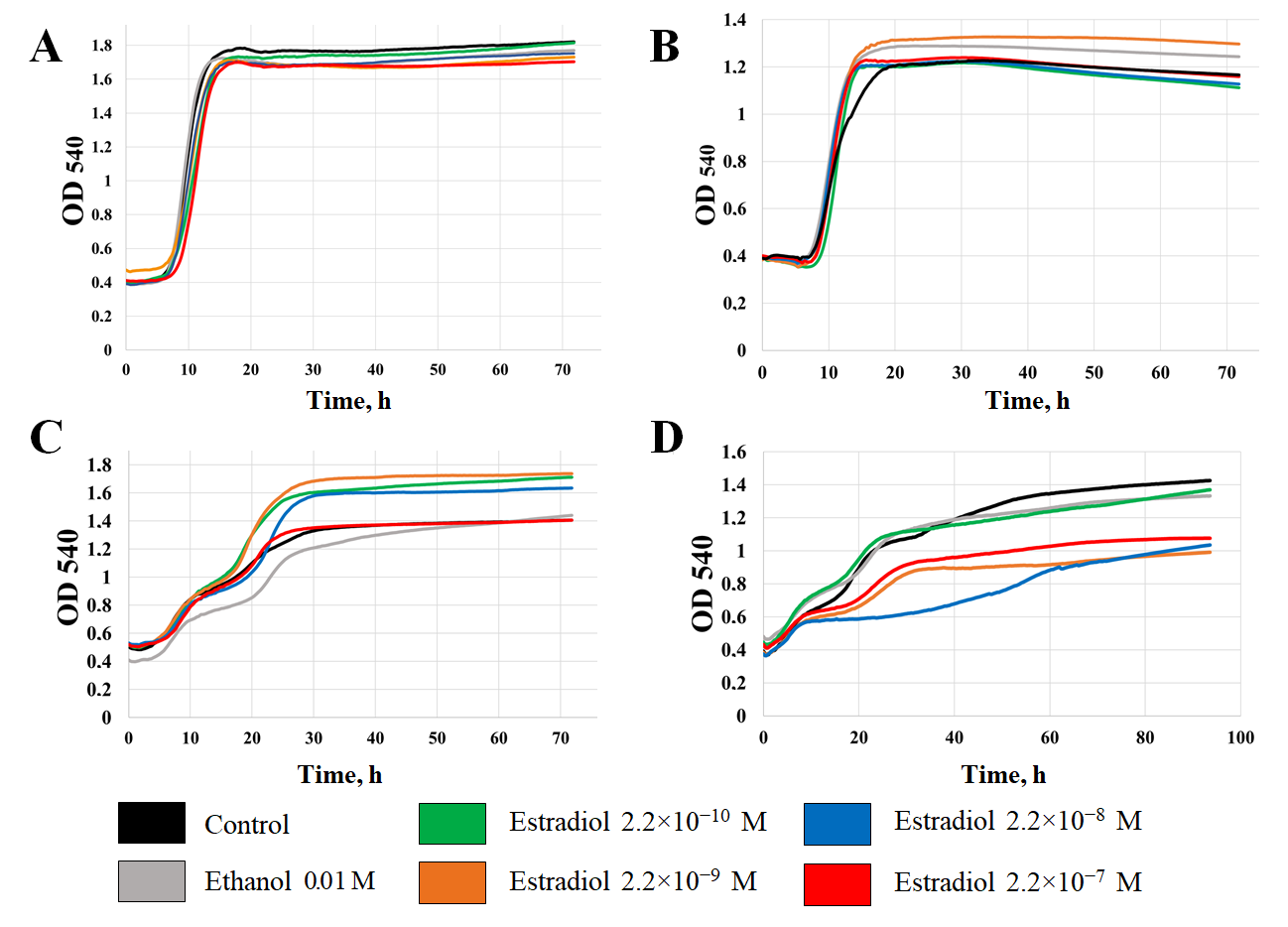

Supplement: Supplementary file 1 [file microorganisms-14-01173-s001.zip › fig-s1-kinetics-estradiol PROOF.png]

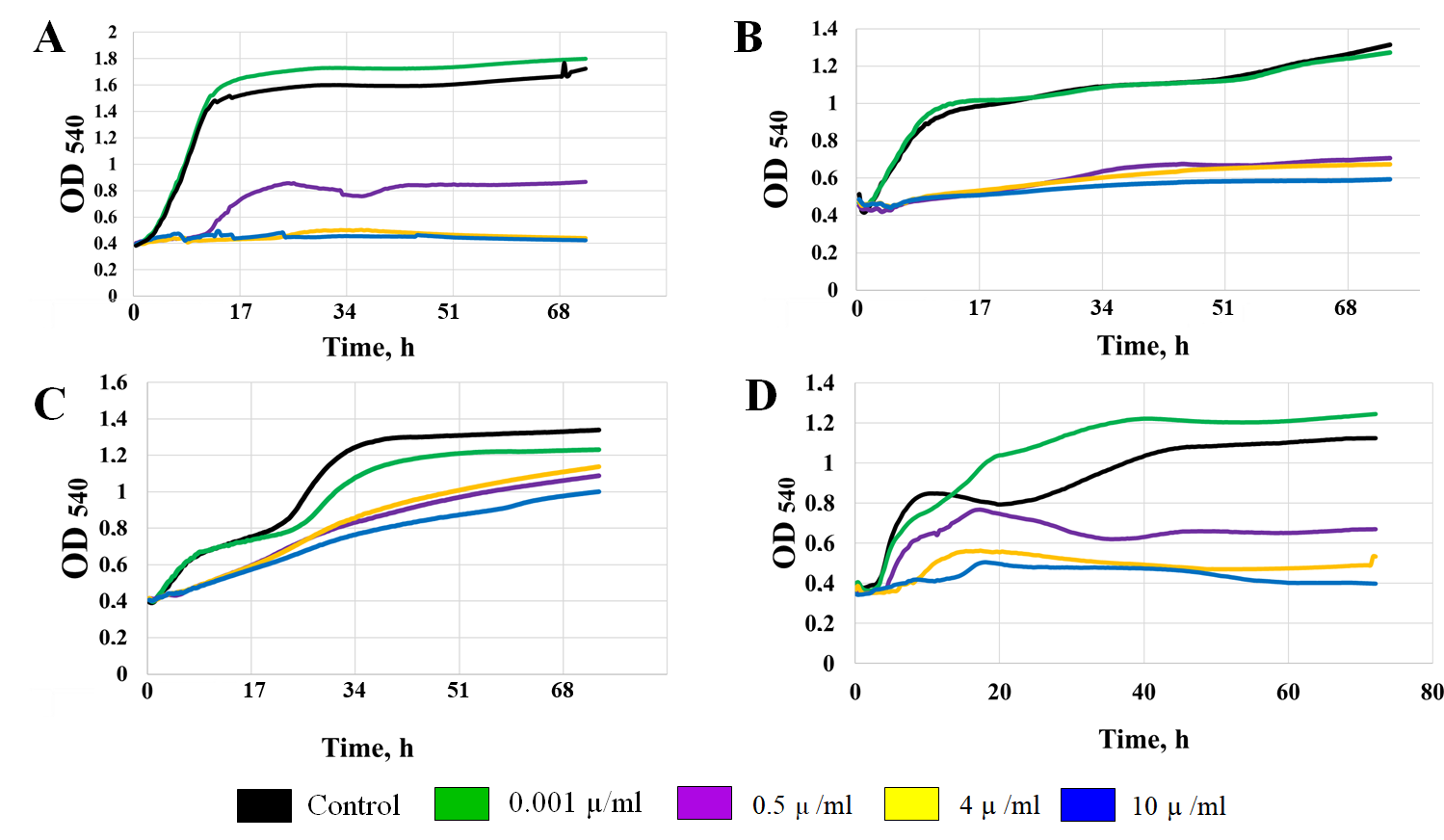

Supplement: Supplementary file 1 [file microorganisms-14-01173-s001.zip › fig-s2-kinetics-clyndamycin PROOF.png]

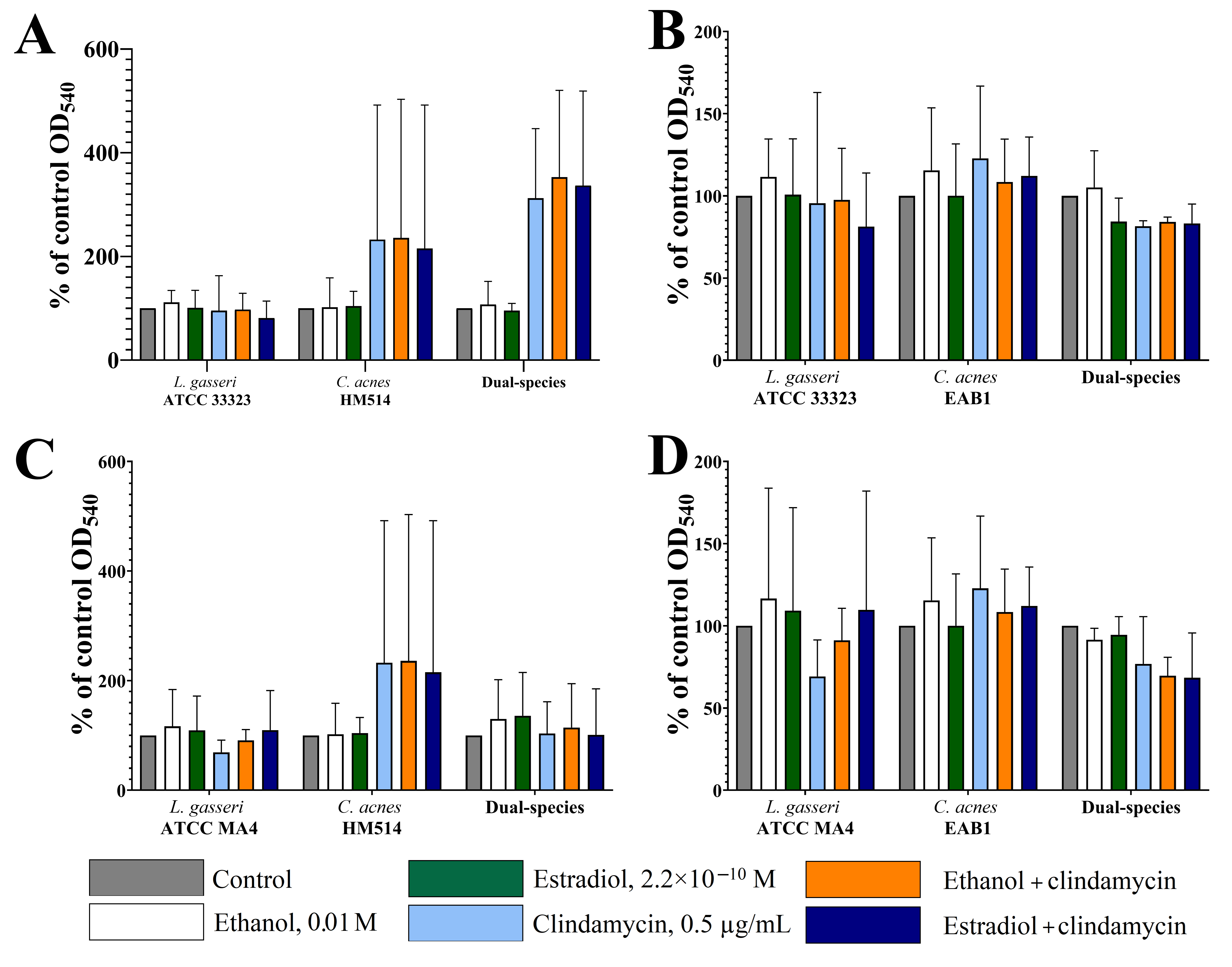

Supplement: Supplementary file 1 [file microorganisms-14-01173-s001.zip › fig-s3-mtt PROOF.png]

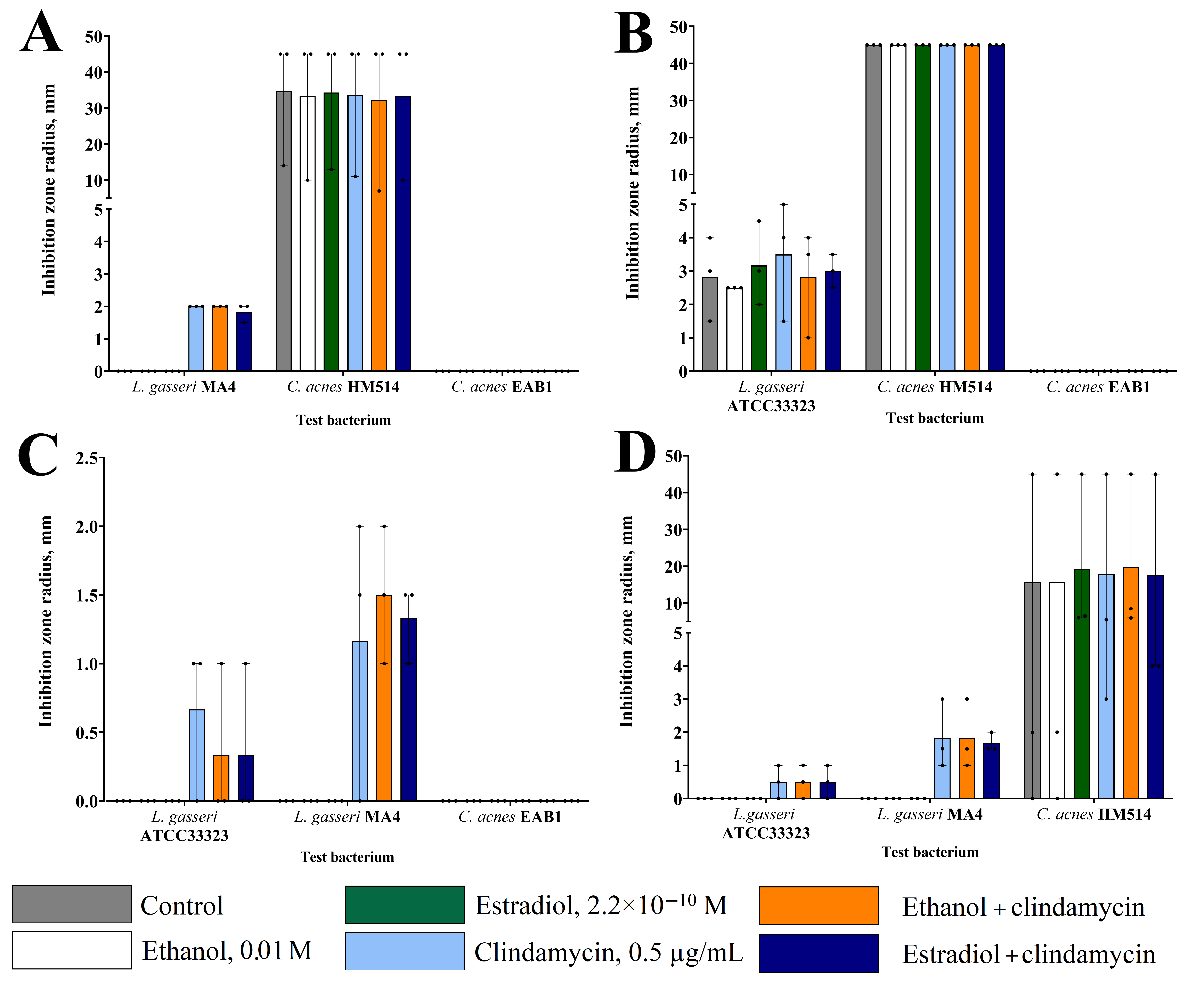

Supplement: Supplementary file 1 [file microorganisms-14-01173-s001.zip › Fig-S4-antagonism-PROOF.png]
